# Fission yeast TOR complex 1 phosphorylates Psk1 through an evolutionarily conserved interaction mediated by the TOS motif

**DOI:** 10.1242/jcs.258865

**Published:** 2021-10-12

**Authors:** Yuichi Morozumi, Ai Hishinuma, Suguru Furusawa, Fajar Sofyantoro, Hisashi Tatebe, Kazuhiro Shiozaki

**Affiliations:** 1 Division of Biological Science, Nara Institute of Science and Technology, Ikoma, Nara 630-0192, Japan; 2 Tohoku Agricultural Research Center, National Agriculture and Food Research Organization, Daisen, Akita 019-2112, Japan; 3 Department of Animal Physiology, Faculty of Biology, Universitas Gadjah Mada, Yogyakarta 55281, Indonesia; 4 Department of Microbiology and Molecular Genetics, University of California, Davis, CA 95616, USA

**Keywords:** TOR complex 1, TORC1, TOS motif, Mip1, Fission yeast

## Abstract

TOR complex 1 (TORC1) is a multi-subunit protein kinase complex that controls cellular growth in response to environmental cues. The regulatory subunits of mammalian TORC1 (mTORC1) include RAPTOR (also known as RPTOR), which recruits mTORC1 substrates, such as S6K1 (also known as RPS6KB1) and 4EBP1 (EIF4EBP1), by interacting with their TOR signaling (TOS) motif. Despite the evolutionary conservation of TORC1, no TOS motif has been described in lower eukaryotes. In the present study, we show that the fission yeast S6 kinase Psk1 contains a TOS motif that interacts with Mip1, a RAPTOR ortholog. The TOS motif in Psk1 resembles those in mammals, including the conserved phenylalanine and aspartic acid residues essential for the Mip1 interaction and TORC1-dependent phosphorylation of Psk1. The binding of the TOS motif to Mip1 is dependent on Mip1 Tyr-533, whose equivalent in RAPTOR is known to interact with the TOS motif in their co-crystals. Furthermore, we utilized the *mip1-Y533A* mutation to screen the known TORC1 substrates in fission yeast and successfully identified Atg13 as a novel TOS-motif-containing substrate. These results strongly suggest that the TOS motif represents an evolutionarily conserved mechanism of the substrate recognition by TORC1.

## Abstract (precis)

**Summary:** By analyzing S6 kinase in fission yeast, we have demonstrated that the TOR signaling (TOS) motif-mediated substrate recognition by TOR complex 1 is conserved from yeast to humans.

## INTRODUCTION

The target of rapamycin (TOR) kinase, a member of the phosphoinositide 3-kinase-related kinase (PIKK) family, is highly conserved among diverse eukaryotes as a master regulator of cell growth and metabolism (Soulard et al., 2009; Wullschleger et al., 2006). TOR forms two distinct multi-subunit complexes termed TOR complex 1 (TORC1) and TOR complex 2 (TORC2), each of which phosphorylates specific sets of cellular substrates in response to diverse stimuli, such as nutrients and growth factors (Laplante and Sabatini, 2012; Saxton and Sabatini, 2017; Zoncu et al., 2011). Mammalian TORC1 (mTORC1), whose regulatory subunits include RAPTOR (also known as RPTOR) (Hara et al., 2002; Kim et al., 2002) and mLST8 (Kim et al., 2003), promotes protein synthesis and other anabolic processes, while suppressing cellular catabolic processes such as autophagy. Accumulating evidence indicates that deregulated activation of mTORC1 is associated with a variety of human diseases, including cancers, diabetes and neurodegenerative disorders (Laplante and Sabatini, 2012; Saxton and Sabatini, 2017; Zoncu et al., 2011).

As the mTOR kinase serves as a catalytic subunit in both mTORC1 and mTORC2, the distinct substrate specificities of the two complexes are likely to be determined by the regulatory subunits unique to each complex. mTORC1 has been proposed to recruit its substrates through the mTORC1-specific subunit RAPTOR. RAPTOR physically interacts with an AGC-family kinase known as ribosomal protein S6 kinase 1 (S6K1, also known as RPS6KB1), as well as eukaryotic initiation factor 4E (eIF4E)-binding protein 1 (4EBP1, also known as EIF4EBP1), two of the best-characterized mTORC1 substrates (Hara et al., 2002; Nojima et al., 2003). The N-terminus of S6K1 and the C-terminus of 4EBP1 contain a consensus sequence of approximately five amino acid residues called the TOR signaling (TOS) motif that binds to RAPTOR. The TOS motif–RAPTOR interaction is essential for the phosphorylation of S6K1 and 4EBP1 by mTORC1 both *in vitro* and *in vivo* (Choi et al., 2003; Nojima et al., 2003; Schalm and Blenis, 2002; Schalm et al., 2003). The proline-rich AKT substrate of 40 kDa (PRAS40, also known as AKT1S1) (Vander Haar et al., 2007; Sancak et al., 2007) also carries the TOS motif, which contributes to the inhibitory interaction of PRAS40 with mTORC1 (Oshiro et al., 2007; Wang et al., 2007). A recent structural study of the co-crystals of RAPTOR and the TOS motif peptides as well as the mTORC1–4EBP1 complex has revealed how the TOS motif interacts with the RAPTOR subunit of mTORC1 (Yang et al., 2017); the TOS motif binds to a groove formed between the RAPTOR N-terminal conserved (RNC) domain and the first several helices of the α-solenoid HEAT repeat in the middle of RAPTOR. On the other hand, no apparent TOS motif is present in many of the known mTORC1 substrates, including MAF1, a negative regulator of RNA polymerase III (Michels et al., 2010) and the autophagy regulators ULK1 and ATG13 (Ganley et al., 2009; Hosokawa et al., 2009; Jung et al., 2009). In contrast to the TOS-motif-dependent recruitment of substrates to mTORC1, very little is known about how mTORC1 recognizes substrates with no discernible TOS motif.

The TORC1 signaling pathway is also conserved in the fission yeast *Schizosaccharomyces pombe* that has been utilized as an excellent model system for the study of diverse cellular processes. Unlike mammalian cells, this unicellular eukaryote has two TOR paralogs named Tor1 and Tor2, which form TORC2 and TORC1, respectively (Morozumi and Shiozaki, 2021; Otsubo and Yamamato, 2008). The regulatory subunits of fission yeast TORC1 include Mip1, a RAPTOR ortholog, as well as the mLST8 equivalent Wat1 (also known as Pop3) (Álvarez and Moreno, 2006; Hayashi et al., 2007; Matsuo et al., 2007). Under nitrogen-replete conditions, activated TORC1 promotes vegetative growth and suppresses sexual differentiation (Álvarez and Moreno, 2006; Matsuo et al., 2007; Uritani et al., 2006). One of the confirmed substrates of fission yeast TORC1 is Psk1, an AGC-family protein kinase responsible for the phosphorylation of the ribosomal S6 proteins in fission yeast (Nakashima et al., 2012); thus, the TORC1-dependent regulation of the S6 protein phosphorylation appears to be conserved from yeast to humans. Similar to the activation of S6K1 by mTORC1 (Magnuson et al., 2012), fission yeast Psk1 is activated by TORC1 through phosphorylation of Thr-392 in the turn motif as well as Thr-415 in the hydrophobic motif (Nakashima et al., 2012). However, no TOS-like motif has been identified in Psk1 or any known TORC1 substrates in lower eukaryotes.

In the present study, we have characterized the physical interaction between Psk1 and the TORC1 subunit Mip1. The RAPTOR ortholog Mip1 interacts with the N-terminus of Psk1, and this interaction is indispensable for the Psk1 phosphorylation by TORC1. Interestingly, the N-terminal region of Psk1 contains an amino acid stretch that resembles the mammalian TOS motif, including the phenylalanine and aspartic acid residues essential for the interaction with RAPTOR. Moreover, the amino acid residues in RAPTOR that are implicated in the direct interaction with the TOS motif are conserved in fission yeast Mip1, and a mutation to one of those residues, *mip1-Y533A*, disrupts its interaction with Psk1. By screening the known TORC1 substrates in the *mip1-Y533A* background, we have successfully identified fission yeast Atg13 as a novel TOS-motif-containing substrate. These results strongly suggest that the mechanism of the TOS-motif-mediated recruitment of the TORC1 substrates is conserved between fission yeast and humans. As far as we know, this study reports the first examples of the TOS motif in lower eukaryotes.

## RESULTS

### A TOS-motif-like sequence in the Psk1 kinase is required for its interaction with Mip1

**Fig. 1.**
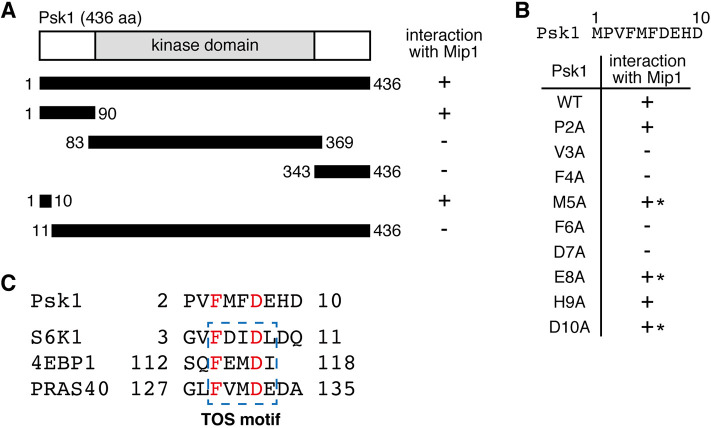
**The N-terminal 10 amino acid region of Psk1 is indispensable for the interaction with Mip1.** (A) Interaction between Mip1 and various Psk1 fragments in yeast two-hybrid assays. Interaction was monitored by *ADE2* and *HIS3* reporter gene expression in the budding yeast Y2HGold strain, as shown in Fig. S1A–C. +, interaction; −, no interaction. (B) Interaction between Mip1 and Psk1 carrying different alanine substitutions in the N-terminal region was examined in yeast two-hybrid assays as in A and Fig. S1D. The amino acid sequence of Psk1 N-terminal region (residues 1–10) is shown at the top. The Psk1 mutants exhibiting milder interaction compared to the Psk1 wild type (WT) are indicated with asterisks. Results in A and B are representative of more than three experiments. (C) Amino acid sequence alignment of the N-terminal region of fission yeast Psk1 and the TOS motif of human S6K1, 4EBP1 and PRAS40. The TOS motifs are shown in a dotted-line box, and the conserved phenylalanine and aspartic acid residues in the TOS motif are shown in red.

Because the RAPTOR subunit of mTORC1 binds and recruits S6K1 for its phosphorylation and activation (see the Introduction), we set out to examine if Mip1, a fission yeast ortholog of RAPTOR, also physically interacts with the TORC1 substrate Psk1. Using Psk1 and Mip1 as bait and prey, respectively, yeast two-hybrid assays were carried out, which successfully detected interaction between the two proteins (Fig. 1A; Fig. S1A). To narrow down the Mip1-binding region within Psk1, yeast two-hybrid assays were repeated with the Psk1 N-terminal fragment (amino acids 1–90), the kinase catalytic domain (amino acids 83–369) and the C-terminal fragment (amino acids 343–436). Among those Psk1 fragments, only the N-terminal fragment of Psk1 exhibited interaction with Mip1 (Fig. 1A; Fig. S1B), and even more strikingly, we found that the N-terminal 10 amino acid residues of Psk1 were sufficient for the interaction (Fig. 1A; Fig. S1C). Consistently, the Psk1 fragment lacking the N-terminal 10 residues (amino acids 11–436) failed to interact with Mip1, indicating that the N-terminal 10 amino acid residues of Psk1 are indispensable for the Psk1–Mip1 interaction.

Next, we assessed each of the Psk1 N-terminal 10 residues for the Mip1 interaction by alanine substitutions of the individual residues followed by yeast two-hybrid assays. Mutations to Val-3, Phe-4, Phe-6, and Asp-7 in Psk1 drastically abolished the interaction with Mip1, whereas those to Met-5, Glu-8 and Asp-10 only partially compromised the Psk1–Mip1 interaction. On the other hand, alanine substitutions of Pro-2 and His-9 showed no apparent impact on the interaction (Fig. 1B; Fig. S1D). We noticed that this N-terminal region of Psk1 is somewhat reminiscent of the TOS motifs in human S6K1, 4EBP1 and PRAS40, with the spaced phenylalanine and aspartic acid residues essential for the interaction with RAPTOR or Mip1 (Fig. 1C; Nojima et al., 2003; Oshiro et al., 2007; Schalm and Blenis, 2002; Schalm et al., 2003; Wang et al., 2007; Yang et al., 2017). Taken together, these results suggest that fission yeast Psk1 has a TOS-motif-like sequence necessary and sufficient for the interaction with the RAPTOR ortholog Mip1.

### The N-terminal TOS motif of Psk1 is essential for its phosphorylation by TORC1

**Fig. 2.**
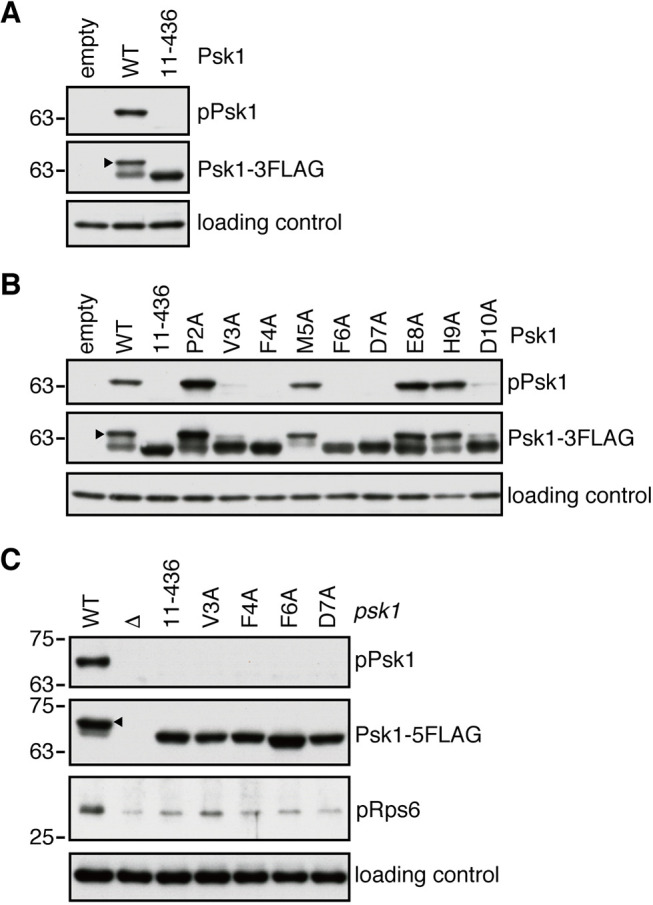
**The TOS-motif-dependent Psk1–Mip1 interaction is essential for the TORC1-dependent phosphorylation of Psk1.** (A) N-terminal 10 amino acid truncation of Psk1 abolished its phosphorylation. A Δ*psk1* strain (CA7589) was transformed with the Psk1 expression plasmid, a derivative of pREP1 plasmid whose *Pnmt1* promoter was replaced with *psk1* endogenous promoter, carrying FLAG-tagged full length Psk1 (wild type, WT) or N-terminal 10 amino acid-truncated Psk1 fragment (11–436). The cell lysate was prepared from the transformants grown in EMM medium at 30°C and analyzed by immunoblotting using anti-FLAG, anti-phospho-S6K1 (pPsk1) and anti-Spc1 (loading control) antibodies. The pREP1 empty vector was used as a negative control (empty). The arrowhead indicates the phosphorylated form of Psk1. (B,C) Psk1 phosphorylation correlates with the ability to binding to Mip1. Fission yeast cells expressing the indicated FLAG-tagged mutant forms of Psk1 from a plasmid (B) or the chromosomal locus (C) were grown in EMM (B) or YES (C) medium at 30°C. The phosphorylation of Psk1 (pPsk1) and Rps6 (pRps6) was visualized by immunoblotting. The arrowhead indicates the phosphorylated form of Psk1. Results are representative of two experiments.

Next, we examined whether the TOS-motif-like stretch found in the N-terminus of Psk1 is required for the TORC1-dependent phosphorylation of Psk1. The *psk1* null (Δ*psk1*) strain was transformed with a plasmid that expresses the full-length or N-terminally truncated (amino acids 11–436) Psk1 with the FLAG epitope tag, followed by immunoblot analysis. As reported previously (Chia et al., 2017; Nakashima et al., 2012), the TORC1-dependent phosphorylation of Thr-415 was readily detected with the full-length Psk1 using the antibodies against phosphorylated S6K1 (‘pPsk1’ in Fig. 2A). Anti-FLAG immunoblotting also showed a slow-migrating band, which corresponds to the phosphorylated form of Psk1 (Chia et al., 2017; Nakashima et al., 2012), confirming the phosphorylation of full-length Psk1. In contrast, these analyses failed to detect phosphorylation of Psk1(11–436) lacking the N-terminal 10 residues (Fig. 2A). Similar experiments were carried out using plasmids that express Psk1 with an alanine substitution of the individual N-terminal residues. We found that the Psk1 phosphorylation was markedly reduced by the V3A, F4A, F6A, D7A and D10A substitutions (Fig. 2B). Consistent results were also obtained when the N-terminal truncation or substitution mutations were introduced to the chromosomal *psk1* gene (Fig. 2C). Moreover, in those mutant strains defective in the Psk1 phosphorylation, the Psk1-dependent phosphorylation of the ribosomal S6 proteins (pRps6 in Fig. 2C) was significantly compromised. These observations are consistent with the earlier report that the TORC1-dependent phosphorylation activates Psk1, which in turn phosphorylates Rps6 (Nakashima et al., 2012). Importantly, those Psk1 mutants with reduced phosphorylation also showed compromised interaction with Mip1 in the yeast two-hybrid assays (Fig. 1A,B); therefore, the Psk1 N-terminal sequence identified by these experiments appears to serve as a TOS motif, which mediates the interaction with the Mip1 subunit of TORC1 as well as the TORC1-dependent phosphorylation and activation of Psk1.

### Mip1 Y533A mutation impairs the TOS-motif-dependent recognition of TORC1 substrates

**Fig. 3.**
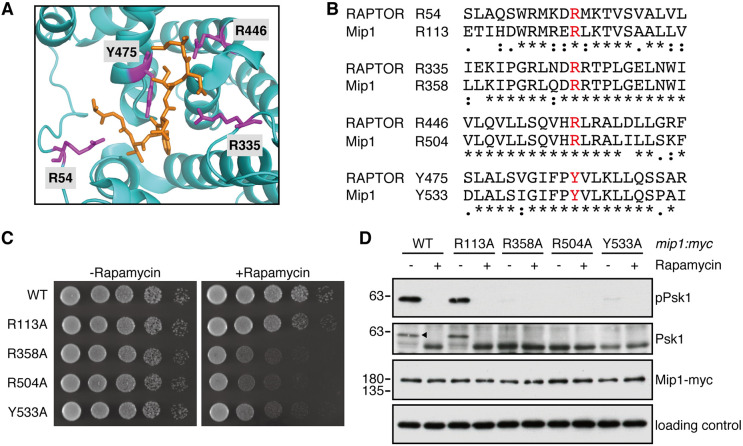
**Alanine substitutions of the Mip1 residues that correspond to the TOS-motif-binding site in RAPTOR result in rapamycin sensitivity and dephosphorylation of Psk1.** (A) Close-up view of the interface between *Arabidopsis thaliana* Raptor (AtRaptor; cyan) and the TOS motif of human S6K1 (orange stick) in the crystal structure of AtRaptor bound to the TOS motif peptide (PDB ID: 5WBK). Side chains of the AtRaptor amino acid residue directly binding to the TOS motif peptide are shown in magenta. Note that all of these AtRaptor residues are conserved in human RAPTOR, whose residue numbers are also shown. (B) Conservation of the amino acid residues (red) that directly interact with the TOS motif between human RAPTOR and fission yeast Mip1. The sequence alignment was performed using the CLUSTALW program (https://www.genome.jp/tools-bin/clustalw). Asterisks indicate identical amino acids. Single and double dots indicate weakly and strongly similar amino acids, respectively. (C) Rapamycin sensitivity of *mip1* mutant strains. Strains carrying the indicated alanine substitutions in *mip1* along with *myc* epitope tag were grown in YES medium at 30°C, and their growth in the presence of rapamycin (100 ng/ml) was tested at 30°C by spotting serial dilutions onto YES agar plates. (D) The strains examined in C were grown in YES medium at 30°C and then treated with either DMSO (negative control) or 200 ng/ml of rapamycin at 30°C for 30 min. The cell lysate was analyzed by immunoblotting using anti*-myc*, anti-phospho-S6K1 (pPsk1), anti-Psk1 and anti-Spc1 (loading control) antibodies. The arrowhead indicates the phosphorylated form of Psk1. Results are representative of two independent experiments. WT, wild type.

Based on the crystal structure of RAPTOR bound to the S6K1 TOS motif peptide, it has been proposed that Arg-54, Arg-335, Arg-446 and Tyr-475 in human RAPTOR directly interact with the TOS motif (Fig. 3A; Yang et al., 2017). Interestingly, all of the four residues are conserved in fission yeast Mip1 (Arg-113, Arg-358, Arg-504 and Tyr-533, respectively; Fig. 3B). To test whether these conserved residues of Mip1 contribute to the interaction with Psk1, we constructed strains whose chromosomal *mip1* gene carries an alanine substitution to each of those residues. The growth rate of all the constructed *mip1* mutants was comparable to those of the wild-type strain (Fig. S2A). We also assessed sexual differentiation of *mip1* mutants; mating of the homothallic (*h^90^*) haploid *mip1* mutants was induced by nitrogen starvation (Fig. S2B), while being suppressed completely on rich yeast extract medium (data not shown), as in the wild-type strain. On the other hand, these *mip1* mutants were sensitive to rapamycin, a specific inhibitor of TORC1, with *mip1-R113A* exhibiting a milder phenotype (Fig. 3C), implying that the TORC1 function is somewhat compromised in these *mip1* mutants. Indeed, the phosphorylation of Psk1 was drastically reduced in these mutant strains with the exception of *mip1-R113A* (Fig. 3D).

**Fig. 4.**
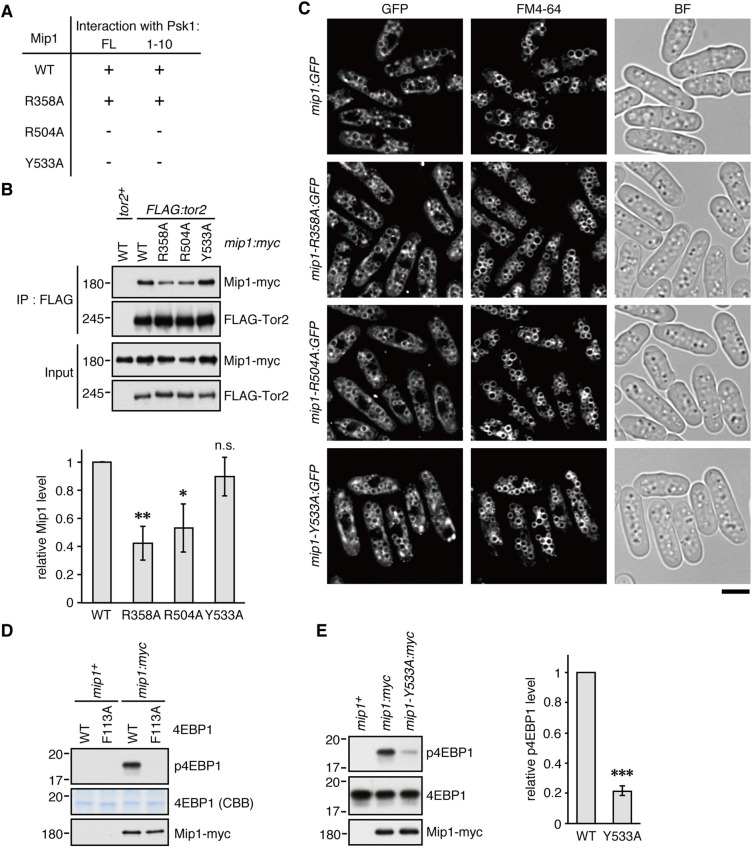
**Alanine substitution of Mip1 Tyr-533 impairs the binding of Mip1 to the TOS motif.** (A) Interaction between wild-type (WT) Mip1 or Mip1 mutants carrying alanine substations at the indicated sites and either Psk1 full length (FL) or N-terminal 10 amino acid region (1–10). Interaction was monitored in a yeast two-hybrid assay by *ADE2* and *HIS3* reporter gene expression in the budding yeast Y2HGold strain, as shown in Fig. S3. +, interaction detected; −, no interaction. Results are representative of four experiments. (B) Integrity of TORC1 containing the Mip1-R358A or Mip1-R504A mutant proteins is partially compromised. FLAG-Tor2 was immunopurified (IP) from the cell lysate of *FLAG:tor2* strains expressing the indicated Mip1*-myc* mutant proteins using anti-FLAG affinity beads, and co-purified Mip1*-myc* was analyzed by immunoblotting. The cell lysate from a strain with the untagged *tor2*^+^ gene was used as a negative control. Mip1 levels in the immunopurified fractions were quantified and shown as value relative to that in the *FLAG-tor2 mip1:myc* strain (mean±s.d., *n*=3 independent experiments). *P<0.05; **P<0.01; n.s., not significant, compared to the wild-type control using one-tailed Student's *t*-test. (C) Alanine substitution of Mip1 residues does not significantly affect the vacuolar localization of Mip1. Fission yeast cells expressing indicated GFP-tagged Mip1 protein from the chromosomal *mip1* locus were grown in EMM at 30°C. The vacuolar membrane was visualized with the fluorescent dye FM4-64. *Z*-axial images were collected, and mid-section images after deconvolution are shown. At least 200 cells were analyzed for each strain. BF, bright-field image. Scale bar: 5 µm. (D) TOS motif-dependent phosphorylation of 4EBP1 by fission yeast TORC1. TORC1 was immunopurified from the cell lysate of the indicated *mip1:myc* strains using anti*-myc* affinity beads. Purified TORC1 was then subjected to a kinase assay using recombinant rat 4EBP1 with and without F113A mutation as substrate, and the reaction mixture was analyzed by immunoblotting using anti-phospho-4EBP1 (T36/45 in rat 4EBP1) and anti*-myc* antibodies. Note that Phe-113 in rat 4EBP1 corresponds to Phe-114 in human 4EBP1 (Fig. 1C). The cell lysate from the untagged *mip1*^+^ strain was used as a negative control. Data are representative of two experiments. CBB, Coomassie Brilliant Blue. (E) TORC1 kinase activity is compromised by alanine substitution of the Mip1 Tyr-533 residue. The kinase activity of TORC1 containing Mip1-Y533A was tested as in D. Quantified p4EBP1 levels relative to that in the *mip1:myc* strain (mean±s.d., *n*=3 independent experiments) are shown as a bar graph on the right. ***P<0.001 (one-tailed Student's *t*-test).

To examine whether the compromised Psk1 phosphorylation in the *mip1-R358A*, *-R504A*, and *-Y533A* mutants is caused by impaired Mip1–Psk1 interaction, we carried out yeast two-hybrid assays using Mip1 carrying those mutations as prey. As expected, Mip1-R504A and Mip1-Y533A hardly interacted with full-length Psk1 as well as its N-terminal fragment of 10 residues (Fig. 4A and Fig. S3). On the other hand, no obvious defect was observed in the interaction between Mip1-R358A and Psk1. One likely possibility is that Mip1-R358A can interact with Psk1 but the mutation affects another function of Mip1, such as its ability to bind the Tor2 kinase for TORC1 assembly. To test the Mip1–Tor2 interaction, we constructed fission yeast strains in which the chromosomal *tor2*^+^ gene is tagged with the FLAG epitope sequence and the wild-type or mutant Mip1 proteins are expressed with the *myc* epitope from the *mip1* locus. Immunoprecipitation of FLAG-tagged Tor2 co-purified comparable amounts of the wild-type and Y533A mutant Mip1 proteins, but less of Mip1-R358A and Mip1-R504A (Fig. 4B). Together, these results suggest that the R358A and R504A substitutions affect the integrity of TORC1, whereas the Y533A mutation more specifically interferes with the recognition of Psk1 by Mip1. We also confirmed that those *mip1* mutations have little impact on the cellular localization of Mip1, by fluorescence microscopy of the wild-type and *mip1* mutant strains with their *mip1* loci tagged with the sequence encoding GFP (Fig. 4C). It was previously reported that fission yeast TORC1 localizes to the surface of vacuoles, the lysosome equivalent in yeasts (Chia et al., 2017; Valbuena et al., 2012); we observed similar vacuolar localization of Mip1-GFP as well as those with the R358A, R504A or Y533A mutations.

Our experiments described above strongly suggest that the impaired phosphorylation (Fig. 3D) and Mip1 interaction (Fig. 4A) of Psk1 in the *mip1-Y533A* mutant are caused by a defect in the evolutionarily conserved mechanism of TOS-motif recognition. To corroborate the defective TOS-motif recognition by Mip1-Y533A, we examined whether TORC1 carrying the Mip1-Y533A subunit fails to phosphorylate the canonical TOS-motif-containing substrate 4EBP1 (Nojima et al., 2003; Schalm et al., 2003). Although 4EBP1 is not conserved in fission yeast, TORC1 isolated from fission yeast cells can phosphorylate rat 4EBP1 *in vitro* (Fig. 4D; Takahara and Maeda, 2012). Importantly, 4EBP1 harboring an alanine substitution of Phe-113 within its TOS motif was not phosphorylated by TORC1 (‘F113A’ in Fig. 4D), demonstrating that fission yeast TORC1 phosphorylates mammalian 4EBP1 in a TOS-motif-dependent manner. As expected, the *in vitro* kinase assay found that TORC1 immunopurified from *mip1-Y533A* cells had significantly compromised activity toward 4EBP1 (Fig. 4E), consistent with the impaired ability of Mip1-Y533A to recognize the TOS motif.

Taken together, these data strongly suggest that the TOS-motif-dependent phosphorylation of TORC1 substrates is conserved between fission yeast and mammals and that the *Y533A* mutation undermines the TOS-motif recognition by the Mip1 subunit.

### *atg13-F538A* mutation impairs TORC1-dependent phosphorylation of Atg13

**Fig. 5.**
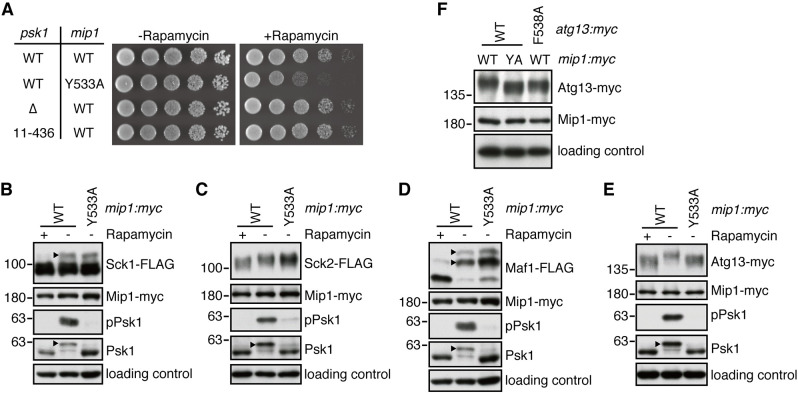
**Alanine substitution of Mip1 Y533 impairs phosphorylation of Atg13, which has a TOS-motif-like sequence.** (A) Loss of Psk1 does not affect cellular sensitivity to rapamycin. Wild-type (WT) and indicated mutant strains were grown in YES medium at 30°C, and their growth in the presence of rapamycin (100 ng/ml) was tested at 30°C by spotting serial dilutions onto YES agar plates. (B–E) The *mip:myc* and *mip1*-*Y533A:myc* strains expressing either FLAG- or myc-tagged TORC1 substrates were grown in YES medium at 30°C, and harvested after DMSO (*mip:myc* and *mip1*-*Y533A:myc* strains) or rapamycin (*mip1:myc* strain) treatment at 30°C for 1 h. The electrophoretic mobilities of Sck1-FLAG (B), Sck2-FLAG (C), Maf1-FLAG (D) and Atg13*-myc* (E) in each condition were compared by immunoblotting. Arrowheads represent the phosphorylated form of the indicated proteins. Note that Maf1-FLAG was analyzed by Phos-tag SDS-PAGE using an 8% SDS polyacrylamide gel containing 50 µM Phos-tag and 100 µM MnCl_2_. (F) The *mip:myc* (WT) and *mip1*-*Y533A:myc* (YA) strains carrying alanine substitution of Atg13 Phe-538 along with *myc* epitope tag were grown in YES medium at 30°C, and their cell lysates were subjected to immunoblot analysis. Data in A–F are representative of more than two experiments.

As shown in Fig. 5A, neither the Δ*psk1* strain nor the *psk1(11-436)* strain defective in Psk1 phosphorylation (Fig. 2) showed any growth defect in the presence or absence of rapamycin. Thus, the TORC1-dependent phosphorylation and activation of the Psk1 kinase (Nakashima et al., 2012) does not appear to be critical to fission yeast cell growth. Therefore, the rapamycin-sensitive phenotype of the *mip1-Y533A* mutant (Figs 3C and 5A) might indicate that phosphorylation of additional TORC1 substrates other than Psk1 is compromised in this mutant. Thus, in *mip1-Y533A* cells we examined the phosphorylation status of Sck1, Sck2, Maf1 and Atg13, the previously reported TORC1 substrates in fission yeast (Du et al., 2012; Nakashima et al., 2012; Otsubo et al., 2017, 2018; Shetty et al., 2020). The FLAG-tagged Sck1 protein phosphorylated by TORC1 is detectable as a band with less electrophoretic mobility (Chia et al., 2017), which disappeared in the presence of rapamycin but remained detectable in the *mip1-Y533A* mutant (Fig. 5B). Similarly, the *mip1-Y533A* mutation exhibited little impact on the slow-migrating, rapamycin-sensitive bands of the Sck2-FLAG (Fig. 5C) and Maf1-FLAG (Fig. 5D) proteins. These observations indicate that the *mip1* mutation does not affect the TORC1-dependent phosphorylation of Sck1, Sck2 and Maf1.

On the other hand, the electrophoretic mobility of Atg13*-myc* increased in wild-type cells treated with rapamycin as well as in the *mip1-Y533A* mutant (Fig. 5E), suggesting impaired TORC1-dependent phosphorylation of Atg13 in the mutant strain. Thus, in addition to Psk1, Atg13 also appears to be phosphorylated by TORC1 in a manner dependent on Mip1 Tyr-533, a residue critical for the TOS-motif-mediated recruitment of TORC1 substrates. Therefore, we carefully scanned the amino acid sequence of Atg13 and found a sequence (FDIDT, amino acids 538–542) with significant similarity to the TOS motif in human S6K1 (Fig. S4). To examine whether this sequence contributes to the TORC1-dependent phosphorylation of Atg13, we constructed a strain whose chromosomal *atg13* gene carries a mutation that substitutes Phe-538 with alanine. Importantly, the electrophoretic mobility of Atg13-F538A protein was found to be comparable to that of the wild-type Atg13 protein in *mip1-Y533A:myc* cells (Fig. 5F). These results strongly suggest that FDIDT in fission yeast Atg13 serves as a TOS motif that mediates the TORC1-dependent phosphorylation of Atg13.

Under nutrient-replete conditions, TORC1 inhibits the initiation of autophagy by phosphorylating the regulatory components of the autophagy pathway, such as Atg13 (Kohda et al., 2007; Otsubo et al., 2017, 2018). As Atg13 phosphorylation is compromised in the *mip1-Y533A* mutant (Fig. 5E), we assessed the induction of autophagy in this mutant by monitoring the proteolytic cleavage of the GFP-Atg8 fusion protein (Mukaiyama et al., 2009). Nitrogen starvation induced the autophagic degradation of GFP-Atg8, releasing free GFP both in wild-type and *mip1-Y533A* cells (‘-N’ in Fig. S5), but not before starvation, suggesting that the *mip1-Y533A* mutation is not enough to induce autophagy. A small amount of the free GFP was detectable in the *mip1-Y533A* mutant treated by rapamycin, whereas rapamycin did not induce autophagy at all in wild-type cells as reported previously (Mukaiyama et al., 2009; Takahara and Maeda, 2012). In addition, the F538A mutation within the TOS-like motif of Atg13 did not affect autophagy induction both in the presence and absence of rapamycin (data not shown). It is likely that the lack of the TOS-motif-dependent phosphorylation of Atg13 per se is insufficient for the autophagy initiation, probably because there are other TORC1 substrates in the negative regulation of autophagy.

## DISCUSSION

In mammals, the RAPTOR subunit serves as a substrate-binding subunit of mTORC1, interacting with the TOS motif in some of the mTORC1 substrates, such as S6K1 and 4EBP1 (Nojima et al., 2003; Schalm and Blenis, 2002). Despite the evolutionary conservation of RAPTOR (Tatebe and Shiozaki, 2017) and the extensive studies of TORC1 signaling in yeast species, there has been no report of the TOS motif in lower eukaryotes. The reported TOS motifs are 5 amino acid residues in length with limited consensus among them, handicapping the sequence-based search for TOS motifs.

In the present study, we have demonstrated that, like S6K1, the fission yeast S6 kinase Psk1 contains a TOS motif in its N-terminus. This short sequence in Psk1 is essential for its interaction with the RAPTOR ortholog Mip1 as well as for its phosphorylation and activation by TORC1. Importantly, the phenylalanine and aspartic acid residues common among the known TOS motifs in the mTORC1 substrates are also conserved in the TOS-motif-like sequence found in Psk1 (Phe-4 and Asp-7; Fig. 1C), and these residues are indispensable for the interaction of Psk1 with Mip1. In the budding yeast *Saccharomyces cerevisiae*, Ypk3 has been identified as an AGC-family kinase that phosphorylates ribosomal protein S6 (González et al., 2015; Yerlikaya et al., 2016). Like human S6K1 and fission yeast Psk1, Ypk3 is phosphorylated and activated by TORC1; however, it remains unknown whether Ypk3 also has a TOS motif. By examining the amino acid sequence of the N-terminal non-catalytic domain of Ypk3, we have found FSLDE (amino acids 3–7), a short sequence that resembles the mammalian TOS motifs as well as the one found in Psk1 (Fig. S4). Although experimental validation is required, we propose that this N-terminal segment of Ypk3 is a strong candidate for the first example of the TOS motif in *S. cerevisiae*.

A co-crystal structural study identified the residues within RAPTOR that directly interact with the TOS motif (Yang et al., 2017), although the contribution of those residues to the substrate recognition by TORC1 *in vivo* has not been evaluated. By mutating the equivalent residues in fission yeast Mip1, we demonstrated that Mip1 Tyr-533, which corresponds to Tyr-475 in RAPTOR, is critical to the interaction of Mip1 with Psk1 as well as the TORC1-dependent phosphorylation of Psk1 *in vivo*. Furthermore, we utilized the *mip1-Y533A* mutation to search for additional TORC1 substrates with a TOS motif and successfully identified Atg13, which indeed contains a TOS-motif-like sequence (FDIDT, amino acids 538–542) essential for its phosphorylation (Fig. S4). We propose that the *mip1-Y533A* mutation characterized in this study and equivalent mutations in RAPTOR and its orthologs can serve as a new tool to identify TORC1 substrates with a TOS motif. Interestingly, *Arabidopsis thaliana* ATG13 possesses a TOS-motif-like sequence (FSDIF, amino acids 189–193), which is important for its interaction with RAPTOR and TORC1-dependent phosphorylation (Fig. S4; Son et al., 2018). On the other hand, we failed to find a TOS-motif-like sequence in human ATG13, possibly because of the short length and limited homology of the TOS motif. Alternatively, human ATG13 may have no TOS motif, being recruited to mTORC1 through a TOS-motif-independent mechanism. It has been reported that ATG13 forms a stable protein complex with ULK1, which is also an mTORC1 substrate that interacts with RAPTOR (Hosokawa et al., 2009). Thus, it is likely that human ATG13 is recruited to mTORC1 by forming a complex with ULK1.

In addition to Atg13, we examined the phosphorylation of Sck1, Sck2 and Maf1, the known TORC1 substrates in fission yeast (Nakashima et al., 2012; Otsubo et al., 2017; Shetty et al., 2020), without detectable effect of the *mip1-Y533A* mutation. It should be noted that budding yeast Maf1 is phosphorylated by both TORC1 (Lee et al., 2009; Wei et al., 2009) and Sch9, an AGC-family kinase activated by TORC1 (Urban et al., 2007). Thus, the rapamycin-sensitive electrophoretic mobility of fission yeast Maf1 (Fig. 5D) may also reflect phosphorylation by not only TORC1 but also an additional kinase(s), making it more difficult to evaluate the effect of *mip1-Y533A* on the Maf1 phosphorylation. In contrast, a previous study demonstrated the TORC1-dependent phosphorylation of Sck1 and Sck2 in fission yeast as well as *in vitro* phosphorylation of purified recombinant Sck1 and Sck2 by TORC1 (Nakashima et al., 2012). Therefore, these AGC-family kinases are likely to be direct substrates of TORC1 but, unlike Psk1, they both failed to interact with Mip1 in our yeast two-hybrid assays (data not shown). We speculate that TORC1 recognizes Sck1 and Sck2 by a TOS-motif-independent mechanism. Indeed, the majority of the known mTORC1 and TORC1 substrates have no apparent TOS motif, and very little is known about how those substrates are specifically recognized by mTORC1 and TORC1.

It has been established that mTORC2 and TORC2 contain the regulatory subunit Sin1, whose conserved region in the middle (CRIM) domain specifically binds and recruits particular AGC-family kinases for phosphorylation and activation (Cameron et al., 2011; Liao and Chen, 2012; Tatebe et al., 2017). Thus, the substrate specificity of the TOR kinase appears to be determined by its evolutionarily conserved regulatory subunits, although the kinase itself is likely to recognize and phosphorylate specific sequences within the recruited substrates. Further studies are required to fully understand the substrate specificity of mTORC1 and TORC1 at a molecular level, particularly the roles of RAPTOR or Mip1 and the other regulatory subunits in the TOS-motif-independent recognition of the substrates.

## MATERIALS AND METHODS

### Fission yeast strains and general methods

*S. pombe* strains used in this study are listed in Table S1. Growth media and basic techniques for *S. pombe* have been described previously (Moreno et al., 1991; Shiozaki and Russell, 1997). For the strain constructions, the PCR-based method was applied to introduce alanine substitutions and the epitope tag sequences to chromosomal genes as reported previously (Bähler et al., 1998; Erdeniz et al., 1997). Each alanine substitution was confirmed by genomic PCR followed by Sanger DNA sequencing.

### Construction of gene expression plasmids

For the plasmids used in yeast two-hybrid assay, various Psk1 and Mip1 fragments were subcloned in the pGBT8 and pGAD GH vectors (Clontech Laboratories), respectively. For the plasmids used in the Psk1 expression experiments in fission yeast, various Psk1 fragments were subcloned in a derivative of pREP1 vector whose *Pnmt1* promoter was replaced with the *psk1*^+^ promoter. The PrimeSTAR Mutagenesis Basal Kit (Takara Bio, Inc.) was used for site-directed mutagenesis.

### Yeast two-hybrid assay

Yeast two-hybrid assays were performed as described previously (Morigasaki et al., 2019). Briefly, the Y2HGold budding yeast strain (Clontech Laboratories) was used as host, and interaction was determined by adenine and histidine auxotrophic markers.

### *S. pombe* growth assay

Fission yeast cells were grown in YES liquid medium, and the cultures were adjusted to the cell concentration equivalent to an optical density at 600 nm (OD_600_) of 1.0. Serial dilutions of the adjusted cultures were spotted onto agar solid media. Images were captured by the LAS-4000 system (Fujifilm, Japan).

### Mating assay

Homothallic haploid *h*^90^ cells were spotted onto a synthetic sporulation agar (SSA) plate (Egel et al., 1974). The cells were analyzed by microscopy after 48 h incubation at 25°C, and the mating efficiency was calculated as described previously (Kunitomo et al., 1995). At least 300 cells were counted for each strain.

### Immunoblotting

Crude cell lysates were prepared using trichloroacetic acid (TCA) as described previously (Tatebe and Shiozaki, 2003). Proteins were separated by SDS-PAGE, transferred to a nitrocellulose membrane, and probed with primary antibodies diluted as follows; anti-phospho-p70 S6K (1:5000; cat. no. 9206, Cell Signaling Technology) for phospho-Psk1 (Thr-415) detection, anti-phospho-Akt substrate (1:4000; cat. no. 9614, Cell Signaling Technology) for phosphorylated Rps6 detection (Nakashima et al., 2010), anti-Psk1 (1:5000; Chia et al., 2017), anti-Spc1 (1:10 000; Tatebe and Shiozaki, 2003), anti-FLAG (1:5000; M2, Sigma-Aldrich), anti*-myc* (1:5000; 9E10, Covance), anti-phospho-4EBP1 (T37/46) (1:2500; cat. no. 2855, Cell Signaling Technology), anti-4EBP1 (1:2500; cat. no. 9452, Cell Signaling Technology), anti-GFP (1:2500; cat. no. 04404, Nacalai Tesque). Anti-rabbit IgG (H+L) HRP-conjugated (1:10 000; cat. no. W4011, Promega), anti-mouse IgG (H+L) HRP-conjugated (1:10 000; cat. no. W4021, Promega) and anti-rat IgG (H+L) HRP-conjugated (1:10 000; cat. no. 112-035-003, Jackson ImmunoResearch) were used as secondary antibodies. For the detection of the phosphorylated form of Maf1, proteins were separated by Phos-tag SDS-PAGE using an 8% SDS polyacrylamide gel containing 50 µM Phos-tag and 100 µM MnCl_2_.

### Preparation of recombinant 4EBP1 expressed in *Escherichia coli*

pET14b expression vector carrying rat 4EBP1 (Addgene #15679; Choi et al., 2003) was transformed into the BL21 codon(+)RIL *E. coli* strain. The cells were grown in LB medium at 37°C until the OD_600_ reached 0.4–0.6. Expression of 4EBP1 was then induced by the addition of 0.1 mM isopropyl-β-D-1-thiogalactopyranoside, and the cells were further cultured at 18°C overnight. The cultures were harvested, resuspended in lysis buffer (50 mM Tris-HCl, pH 7.5, 500 mM NaCl, 2 mM 2-mercaptoethanol and 10% glycerol) and disrupted by sonication (for 10 min with on-time of 1 s and off-time of 1 s at 4°C). The cell debris was removed by centrifugation for 20 min at 10 000 ***g***, and the supernatant was mixed gently with 1 ml of Ni-NTA agarose beads (Fujifilm Wako) at 4°C for 1 h. The protein-bound beads were packed into an Econo-Column (Bio-Rad Laboratories) and washed with lysis buffer. The proteins were eluted with lysis buffer containing 500 mM imidazole and dialyzed against storage buffer (20 mM Tris-HCl, pH 7.5, 200 mM NaCl, 2 mM 2-mercaptoethanol and 10% glycerol). The samples were flash-frozen in liquid nitrogen and stored at −80°C.

### Immunoprecipitation and *in vitro* kinase assay

Yeast cells were disrupted in lysis buffer (20 mM HEPES-NaOH, pH 7.5, 150 mM sodium glutamate, 10% glycerol, 0.25% Tween-20, 10 mM sodium fluoride, 10 mM p-nitrophenylphosphate, 10 mM sodium pyrophosphate, 10 mM β-glycerophosphate and 0.1 mM sodium orthovanadate) containing 1 mM phenylmethylsulfonyl fluoride and protease inhibitor cocktail (P8849, Sigma-Aldrich) with glass beads using a Multi-beads Shocker (Yasui Kikai). Cell lysate was recovered by centrifugation for 15 min at 17 700 ***g*** and the total protein concentrations of cell lysates were determined by Bradford assay. For interaction between FLAG-tagged protein and *myc*-tagged protein, the recovered cell lysates were incubated with anti-FLAG M2-affinity gel (Sigma-Aldrich) for 2 h at 4°C, followed by extensive washing with lysis buffer. Resultant samples were subjected to immunoblotting.

For the *in vitro* kinase assay, cell lysates prepared from a fission yeast *mip1:myc* strain were incubated with anti-c*-myc* antibody beads (Fujifilm Wako) at 4°C for 2 h. The beads were washed with lysis buffer followed by additional washing with kinase wash buffer [20 mM HEPES-NaOH, pH 7.5, 150 mM NaCl and 1 mM dithiothreitol (DTT)]. The beads were then mixed with 250 ng of rat 4EBP1 in 20 μl of reaction buffer (20 mM HEPES-NaOH, pH 7.5, 1 mM MgCl_2_, 1 mM DTT and 100 mM NaCl) and reactions were initiated by the addition of ATP (1 mM). After incubation at 30°C for 30 min, the reactions were stopped by addition of Laemmli sample buffer, and the phosphorylation of 4EBP1 was monitored by immunoblotting.

### Microscopy

Fluorescence microscopic analysis was performed using a DeltaVision Elite Microscopy System (GE Healthcare) as described previously (Chia et al., 2017; Morigasaki et al., 2019). Briefly, cells grown exponentially in EMM liquid were stained with FM4-64 fluorescent dye (Fujifilm Wako) for vacuole visualization and mounted on a thin layer of EMM agar. Z-axial images were taken at 0.4 μm with a 60× objective lens. Deconvolution of images was performed using DeltaVision SoftWoRx software.

## Supplementary Material

Supplementary information

Reviewer comments

## References

[JCS258865C1] Álvarez, B. and Moreno, S. (2006). Fission yeast Tor2 promotes cell growth and represses cell differentiation. *J. Cell Sci.* 119, 4475-4485. 10.1242/jcs.03241 17046992

[JCS258865C2] Bähler, J., Wu, J.-Q., Longtine, M. S., Shah, N. G., Mckenzie, A., III, Steever, A. B., Wach, A., Philippsen, P. and Pringle, J. R. (1998). Heterologous modules for efficient and versatile PCR-based gene targeting in Schizosaccharomyces pombe. *Yeast* 14, 943-951. 10.1002/(SICI)1097-0061(199807)14:10<943::AID-YEA292>3.0.CO;2-Y 9717240

[JCS258865C3] Cameron, A. J. M., Linch, M. D., Saurin, A. T., Escribano, C. and Parker, P. J. (2011). mTORC2 targets AGC kinases through Sin1-dependent recruitment. *Biochem. J.* 439, 287-297. 10.1042/BJ20110678 21806543

[JCS258865C4] Chia, K. H., Fukuda, T., Sofyantoro, F., Matsuda, T., Amai, T. and Shiozaki, K. (2017). Ragulator and GATOR1 complexes promote fission yeast growth by attenuating TOR complex 1 through Rag GTPases. *eLife* 6, e30880. 10.7554/eLife.30880 29199950 PMC5752196

[JCS258865C5] Choi, K. M., McMahon, L. P. and Lawrence, J. C. (2003). Two Motifs in the translational repressor PHAS-I required for efficient phosphorylation by mammalian target of rapamycin and for recognition by raptor. *J. Biol. Chem.* 278, 19667-19673. 10.1074/jbc.M301142200 12665511

[JCS258865C6] Du, W., Hálová, L., Kirkham, S., Atkin, J. and Petersen, J. (2012). TORC2 and the AGC kinase Gad8 regulate phosphorylation of the ribosomal protein S6 in fission yeast. *Biol. Open* 1, 884-888. 10.1242/bio.20122022 23213482 PMC3507231

[JCS258865C57] Egel, R. and Egel-Mitani, M. (1974). Premeiotic DNA synthesis in fission yeast. *Exp. Cell Res.* 88, 127-134. 10.1016/0014-4827(74)90626-0 4472733

[JCS258865C7] Erdeniz, N., Mortensen, U. H. and Rothstein, R. (1997). Cloning-free PCR-based allele replacement methods. *Genome Res.* 7, 1174-1183. 10.1101/gr.7.12.1174 9414323 PMC310678

[JCS258865C8] Ganley, I. G., Lam, D. H., Wang, J., Ding, X., Chen, S. and Jiang, X. (2009). ULK1·ATG13·FIP200 complex mediates mTOR signaling and is essential for autophagy. *J. Biol. Chem.* 284, 12297-12305. 10.1074/jbc.M900573200 19258318 PMC2673298

[JCS258865C9] González, A., Shimobayashi, M., Eisenberg, T., Merle, D. A., Pendl, T., Hall, M. N. and Moustafa, T. (2015). TORC1 promotes phosphorylation of ribosomal protein S6 via the AGC Kinase Ypk3 in Saccharomyces cerevisiae. *PLoS ONE* 10, e0120250. 10.1371/journal.pone.0120250 25767889 PMC4359079

[JCS258865C10] Hara, K., Maruki, Y., Long, X., Yoshino, K.-i., Oshiro, N., Hidayat, S., Tokunaga, C., Avruch, J. and Yonezawa, K. (2002). Raptor, a binding partner of Target of Rapamycin (TOR), mediates TOR action. *Cell* 110, 177-189. 10.1016/S0092-8674(02)00833-4 12150926

[JCS258865C11] Hayashi, T., Hatanaka, M., Nagao, K., Nakaseko, Y., Kanoh, J., Kokubu, A., Ebe, M. and Yanagida, M. (2007). Rapamycin sensitivity of the Schizosaccharomyces pombe tor2 mutant and organization of two highly phosphorylated TOR complexes by specific and common subunits. *Genes Cells* 12, 1357-1370. 10.1111/j.1365-2443.2007.01141.x 18076573

[JCS258865C12] Hosokawa, N., Hara, T., Kaizuka, T., Kishi, C., Takamura, A., Miura, Y., Iemura, S.-i., Natsume, T., Takehana, K., Yamada, N. et al. (2009). Nutrient-dependent mTORC1 association with the ULK1–Atg13–FIP200 complex required for autophagy. *Mol. Biol. Cell* 20, 1981-1991. 10.1091/mbc.e08-12-1248 19211835 PMC2663915

[JCS258865C13] Jung, C. H., Jun, C. B., Ro, S.-H., Kim, Y.-M., Otto, N. M., Cao, J., Kundu, M. and Kim, D.-H. (2009). ULK-Atg13-FIP200 complexes mediate mTOR signaling to the autophagy machinery. *Mol. Biol. Cell* 20, 1992-2003. 10.1091/mbc.e08-12-1249 19225151 PMC2663920

[JCS258865C14] Kim, D.-H., Sarbassov, D. D., Ali, S. M., King, J. E., Latek, R. R., Erdjument-Bromage, H., Tempst, P. and Sabatini, D. M. (2002). mTOR interacts with raptor to form a nutrient-sensitive complex that signals to the cell growth machinery. *Cell* 110, 163-175. 10.1016/S0092-8674(02)00808-5 12150925

[JCS258865C15] Kim, D.-H., Sarbassov, D. D., Ali, S. M., Latek, R. R., Guntur, K. V. P., Erdjument-Bromage, H., Tempst, P. and Sabatini, D. M. (2003). GβL, a positive regulator of the rapamycin-sensitive pathway required for the nutrient-sensitive interaction between raptor and mTOR. *Mol. Cell* 11, 895-904. 10.1016/S1097-2765(03)00114-X 12718876

[JCS258865C16] Kohda, T. A., Tanaka, K., Konomi, M., Sato, M., Osumi, M. and Yamamoto, M. (2007). Fission yeast autophagy induced by nitrogen starvation generates a nitrogen source that drives adaptation processes. *Genes Cells* 12, 155-170. 10.1111/j.1365-2443.2007.01041.x 17295836

[JCS258865C17] Kunitomo, H., Sugimoto, A., Yamamoto, M. and Wilkinson, C. R. M. (1995). Schizosaccharomyces pombe pac2+controls the onset of sexual development via a pathway independent of the cAMP cascade. *Curr. Genet.* 28, 32-38. 10.1007/BF00311879 8536311

[JCS258865C18] Laplante, M. and Sabatini, D. M. (2012). mTOR signaling in growth control and disease. *Cell* 149, 274-293. 10.1016/j.cell.2012.03.017 22500797 PMC3331679

[JCS258865C19] Lee, J., Moir, R. D. and Willis, I. M. (2009). Regulation of RNA polymerase III transcription involves SCH9-dependent and SCH9-independent branches of the Target of Rapamycin (TOR) pathway. *J. Biol. Chem.* 284, 12604-12608. 10.1074/jbc.C900020200 19299514 PMC2675989

[JCS258865C20] Liao, H.-C. and Chen, M.-Y. (2012). Target of rapamycin complex 2 signals to downstream effector yeast protein kinase 2 (Ypk2) through adheres-voraciously-to-target-of-rapamycin-2 protein 1 (Avo1) in Saccharomyces cerevisiae. *J. Biol. Chem.* 287, 6089-6099. 10.1074/jbc.M111.303701 22207764 PMC3307311

[JCS258865C21] Magnuson, B., Ekim, B. and Fingar, D. C. (2012). Regulation and function of ribosomal protein S6 kinase (S6K) within mTOR signalling networks. *Biochem. J.* 441, 1-21. 10.1042/BJ20110892 22168436

[JCS258865C22] Matsuo, T., Otsubo, Y., Urano, J., Tamanoi, F. and Yamamoto, M. (2007). Loss of the TOR Kinase Tor2 mimics nitrogen starvation and activates the sexual development pathway in fission yeast. *Mol. Cell. Biol.* 27, 3154-3164. 10.1128/MCB.01039-06 17261596 PMC1899950

[JCS258865C23] Michels, A. A., Robitaille, A. M., Buczynski-Ruchonnet, D., Hodroj, W., Reina, J. H., Hall, M. N. and Hernandez, N. (2010). mTORC1 directly phosphorylates and regulates human MAF1. *Mol. Cell. Biol.* 30, 3749-3757. 10.1128/MCB.00319-10 20516213 PMC2916396

[JCS258865C24] Moreno, S., Klar, A. and Nurse, P. (1991). Molecular genetic analysis of fission yeast Schizosaccharomyces pombe. *Methods Enzymol.* 194, 795-823. 10.1016/0076-6879(91)94059-L 2005825

[JCS258865C25] Morigasaki, S., Chin, L. C., Hatano, T., Emori, M., Iwamoto, M., Tatebe, H. and Shiozaki, K. (2019). Modulation of TOR complex 2 signaling by the stress-activated MAPK pathway in fission yeast. *J. Cell Sci.* 132, jcs236133. 10.1242/jcs.236133 31477575

[JCS258865C26] Morozumi, Y. and Shiozaki, K. (2021). Conserved and divergent mechanisms that control torc1 in yeasts and mammals. *Genes* 12, 88. 10.3390/genes12010088 33445779 PMC7828246

[JCS258865C27] Mukaiyama, H., Kajiwara, S., Hosomi, A., Giga-Hama, Y., Tanaka, N., Nakamura, T. and Takegawa, K. (2009). Autophagy-deficient Schizosaccharomyces pombe mutants undergo partial sporulation during nitrogen starvation. *Microbiology* 155, 3816-3826. 10.1099/mic.0.034389-0 19778961

[JCS258865C28] Nakashima, A., Sato, T. and Tamanoi, F. (2010). Fission yeast TORC1 regulates phosphorylation of ribosomal S6 proteins in response to nutrients and its activity is inhibited by rapamycin. *J. Cell Sci.* 123, 777-786. 10.1242/jcs.060319 20144990 PMC2823578

[JCS258865C29] Nakashima, A., Otsubo, Y., Yamashita, A., Sato, T., Yamamoto, M. and Tamanoi, F. (2012). Psk1, an AGC kinase family member in fission yeast, is directly phosphorylated and controlled by TORC1 and functions as S6 kinase. *J. Cell Sci.* 125, 5840-5849. 10.1242/jcs.111146 22976295 PMC3575713

[JCS258865C30] Nojima, H., Tokunaga, C., Eguchi, S., Oshiro, N., Hidayat, S., Yoshino, K.-i., Hara, K., Tanaka, N., Avruch, J. and Yonezawa, K. (2003). The Mammalian Target of Rapamycin (mTOR) partner, raptor, binds the mTOR Substrates p70 S6 Kinase and 4E-BP1 through their TOR Signaling (TOS) motif. *J. Biol. Chem.* 278, 15461-15464. 10.1074/jbc.C200665200 12604610

[JCS258865C31] Oshiro, N., Takahashi, R., Yoshino, K.-i., Tanimura, K., Nakashima, A., Eguchi, S., Miyamoto, T., Hara, K., Takehana, K., Avruch, J. et al. (2007). The Proline-rich Akt Substrate of 40 kDa (PRAS40) is a physiological substrate of mammalian target of rapamycin complex 1. *J. Biol. Chem.* 282, 20329-20339. 10.1074/jbc.M702636200 17517883 PMC3199301

[JCS258865C32] Otsubo, Y. and Yamamato, M. (2008). TOR signaling in fission yeast. *Crit. Rev. Biochem. Mol. Biol.* 43, 277-283. 10.1080/10409230802254911 18756382

[JCS258865C33] Otsubo, Y., Nakashima, A., Yamamoto, M. and Yamashita, A. (2017). TORC1-dependent phosphorylation targets in fission yeast. *Biomolecules* 7, 50. 10.3390/biom7030050 PMC5618231 28671615

[JCS258865C34] Otsubo, Y., Matsuo, T., Nishimura, A., Yamamoto, M. and Yamashita, A. (2018). tRNA production links nutrient conditions to the onset of sexual differentiation through the TORC1 pathway. *EMBO Rep.* 19, e44867. 10.15252/embr.201744867 29330317 PMC5836105

[JCS258865C35] Sancak, Y., Thoreen, C. C., Peterson, T. R., Lindquist, R. A., Kang, S. A., Spooner, E., Carr, S. A. and Sabatini, D. M. (2007). PRAS40 is an insulin-regulated inhibitor of the mTORC1 protein kinase. *Mol. Cell* 25, 903-915. 10.1016/j.molcel.2007.03.003 17386266

[JCS258865C36] Saxton, R. A. and Sabatini, D. M. (2017). mTOR signaling in growth, metabolism, and disease. *Cell* 168, 960-976. 10.1016/j.cell.2017.02.004 28283069 PMC5394987

[JCS258865C37] Schalm, S. S. and Blenis, J. (2002). Identification of a conserved motif required for mTOR signaling. *Curr. Biol.* 12, 632-639. 10.1016/S0960-9822(02)00762-5 11967149

[JCS258865C38] Schalm, S. S., Fingar, D. C., Sabatini, D. M. and Blenis, J. (2003). TOS motif-mediated raptor binding regulates 4E-BP1 multisite phosphorylation and function. *Curr. Biol.* 13, 797-806. 10.1016/S0960-9822(03)00329-4 12747827

[JCS258865C39] Shetty, M., Noguchi, C., Wilson, S., Martinez, E., Shiozaki, K., Sell, C., Mell, J. C. and Noguchi, E. (2020). Maf1-dependent transcriptional regulation of tRNAs prevents genomic instability and is associated with extended lifespan. *Aging Cell* 19, e13068. 10.1111/acel.13068 31833215 PMC6996946

[JCS258865C40] Shiozaki, K. and Russell, P. (1997). Stress-activated protein kinase pathway in cell cycle control of fission yeast. *Methods Enzymol.* 283, 506-520. 10.1016/S0076-6879(97)83040-6 9251044

[JCS258865C41] Son, O., Kim, S., Kim, D., Hur, Y.-S., Kim, J. and Cheon, C.-I. (2018). Involvement of TOR signaling motif in the regulation of plant autophagy. *Biochem. Biophys. Res. Commun.* 501, 643-647. 10.1016/j.bbrc.2018.05.027 29738770

[JCS258865C42] Soulard, A., Cohen, A. and Hall, M. N. (2009). TOR signaling in invertebrates. *Curr. Opin. Cell Biol.* 21, 825-836. 10.1016/j.ceb.2009.08.007 19767189

[JCS258865C43] Takahara, T. and Maeda, T. (2012). TORC1 of fission yeast is rapamycin-sensitive. *Genes Cells* 17, 698-708. 10.1111/j.1365-2443.2012.01618.x 22762302

[JCS258865C44] Tatebe, H. and Shiozaki, K. (2003). Identification of Cdc37 as a novel regulator of the stress-responsive mitogen-activated protein kinase. *Mol. Cell. Biol.* 23, 5132-5142. 10.1128/MCB.23.15.5132-5142.2003 12861001 PMC165716

[JCS258865C45] Tatebe, H. and Shiozaki, K. (2017). Evolutionary conservation of the components in the TOR signaling pathways. *Biomolecules* 7, 77. 10.3390/biom7040077 PMC5745459 29104218

[JCS258865C46] Tatebe, H., Murayama, S., Yonekura, T., Hatano, T., Richter, D., Furuya, T., Kataoka, S., Furuita, K., Kojima, C. and Shiozaki, K. (2017). Substrate specificity of tor complex 2 is determined by a ubiquitin-fold domain of the sin1 subunit. *eLife* 6, e19594. 10.7554/eLife.19594 28264193 PMC5340527

[JCS258865C47] Urban, J., Soulard, A., Huber, A., Lippman, S., Mukhopadhyay, D., Deloche, O., Wanke, V., Anrather, D., Ammerer, G., Riezman, H. et al. (2007). Sch9 Is a Major Target of TORC1 in Saccharomyces cerevisiae. *Mol. Cell* 26, 663-674. 10.1016/j.molcel.2007.04.020 17560372

[JCS258865C48] Uritani, M., Hidaka, H., Hotta, Y., Ueno, M., Ushimaru, T. and Toda, T. (2006). Fission yeast Tor2 links nitrogen signals to cell proliferation and acts downstream of the Rheb GTPase. *Genes Cells* 11, 1367-1379. 10.1111/j.1365-2443.2006.01025.x 17121544

[JCS258865C49] Valbuena, N., Rozalén, A. E. and Moreno, S. (2012). Fission yeast TORC1 prevents eIF2α phosphorylation in response to nitrogen and amino acids via Gcn2 kinase. *J. Cell Sci.* 125, 5955-5959. 10.1242/jcs.105395 23108671

[JCS258865C50] Vander Haar, E., Lee, S.-i., Bandhakavi, S., Griffin, T. J. and Kim, D.-H. (2007). Insulin signalling to mTOR mediated by the Akt/PKB substrate PRAS40. *Nat. Cell Biol.* 9, 316-323. 10.1038/ncb1547 17277771

[JCS258865C51] Wang, L., Harris, T. E., Roth, R. A. and Lawrence, J. C. (2007). PRAS40 regulates mTORC1 kinase activity by functioning as a direct inhibitor of substrate binding. *J. Biol. Chem.* 282, 20036-20044. 10.1074/jbc.M702376200 17510057

[JCS258865C52] Wei, Y., Tsang, C. K. and Zheng, X. F. S. (2009). Mechanisms of regulation of RNA polymerase III-dependent transcription by TORC1. *EMBO J.* 28, 2220-2230. 10.1038/emboj.2009.179 19574957 PMC2726700

[JCS258865C53] Wullschleger, S., Loewith, R. and Hall, M. N. (2006). TOR signaling in growth and metabolism. *Cell* 124, 471-484. 10.1016/j.cell.2006.01.016 16469695

[JCS258865C54] Yang, H., Jiang, X., Li, B., Yang, H. J., Miller, M., Yang, A., Dhar, A. and Pavletich, N. P. (2017). Mechanisms of mTORC1 activation by RHEB and inhibition by PRAS40. *Nature* 552, 368-373. 10.1038/nature25023 29236692 PMC5750076

[JCS258865C55] Yerlikaya, S., Meusburger, M., Kumari, R., Huber, A., Anrather, D., Costanzo, M., Boone, C., Ammerer, G., Baranov, P. V. and Loewith, R. (2016). TORC1 and TORC2 work together to regulate ribosomal protein S6 phosphorylation in Saccharomyces cerevisiae. *Mol. Biol. Cell* 27, 397-409. 10.1091/mbc.e15-08-0594 26582391 PMC4713140

[JCS258865C56] Zoncu, R., Efeyan, A. and Sabatini, D. M. (2011). mTOR: from growth signal integration to cancer, diabetes and ageing. *Nat. Rev. Mol. Cell Biol.* 12, 21-35. 10.1038/nrm3025 21157483 PMC3390257

