# Supplementary material for: Fission yeast TOR complex 1 phosphorylates Psk1 through an evolutionarily conserved interaction mediated by the TOS motif
Source: J Cell Sci. 2021 Oct 12;134(19):jcs258865. doi: 10.1242/jcs.258865 (PMC8542387; doi:10.1242/jcs.258865)
Supplement: Supplementary information [file joces-134-258865-s1.pdf]

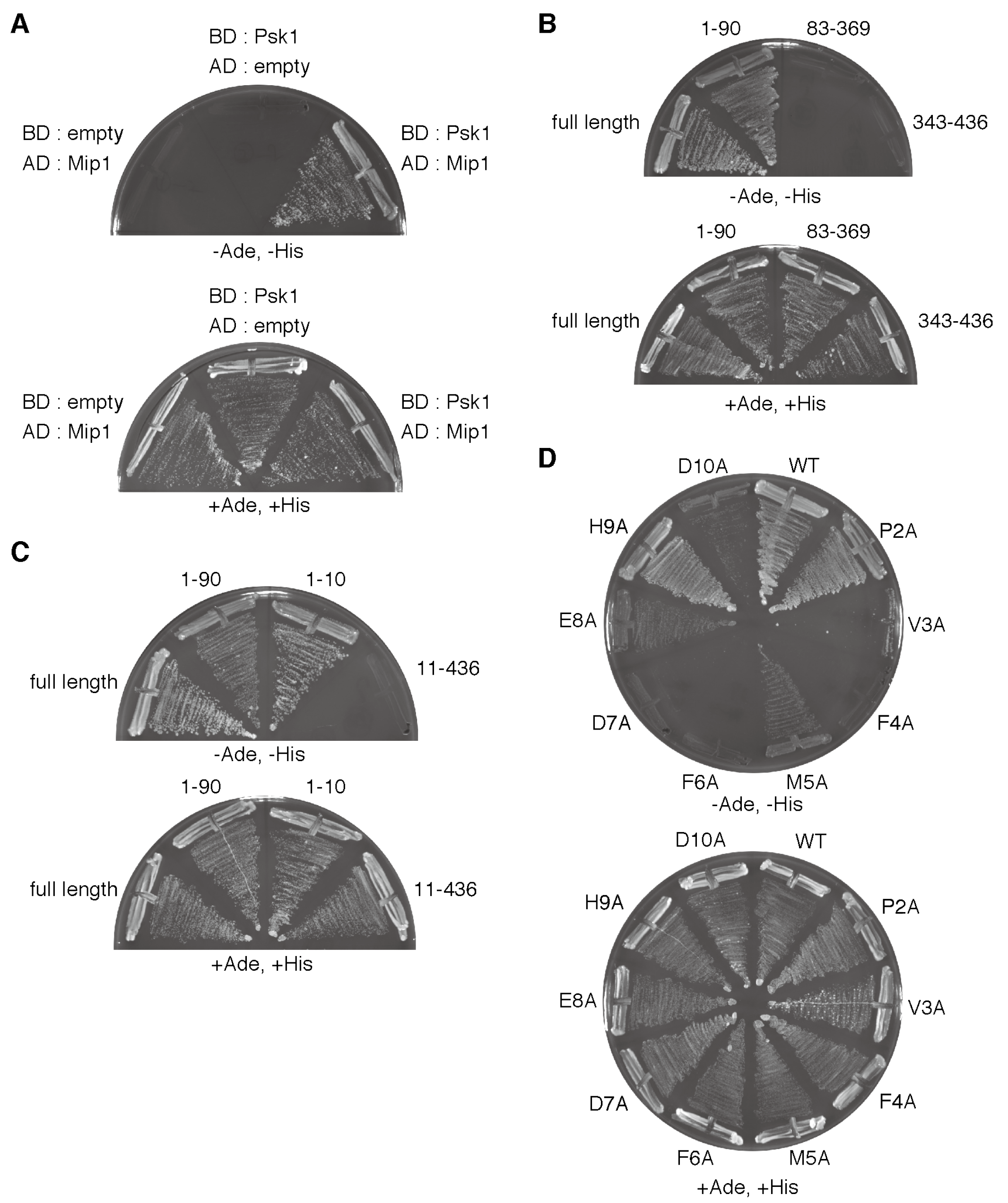

Fig. S1. Characterization of the interaction between Mip1 and Psk1 by yeast two-hybrid assays (related to Figure 1).

(A) Psk1 and Mip1 fused to Gal4 DNA-binding (BD) and activation (AD) domains, respectively, were expressed in the budding yeast Y2HGold, and their interaction was judged by adenine and histidine auxotrophy. Cells carrying either of the empty vectors were used as negative controls. (B, C) Interactions between Mip1 and various Psk1 fragments were tested as in (A). (D) Interactions between Mip1 and the Psk1 mutants harboring the indicated alanine substitutions in its N-terminal region were tested as in (A).

Journal of Cell Science • Supplementary information

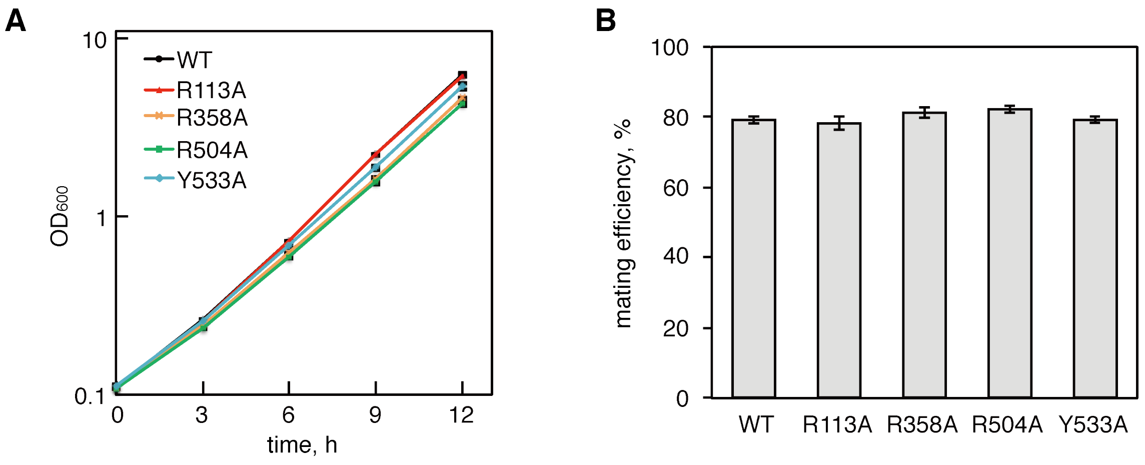

Fig. S2. The alanine substitution mutations of *mip1* used in this study have little impact on cell proliferation and sexual differentiation.

(A) The indicated *mip1* mutant strains were grown in YES medium at 30˚C, and their growth was monitored by measuring OD_{600} every 3 hours. Results are shown as means±s.d. from three independent experiments. Black, wild-type; red, *mip1-R113A*; orange, *mip1-R358A*; green, *mip1-R504A*; cyan, *mip1-Y533A*. (B) Homothallic h^{90} strains of the indicated mutants were incubated on SSA sporulation agar meidum at 25°C for 48 hours, and their mating efficiency was monitored. Means±s.d. from three independent experiments are presented.

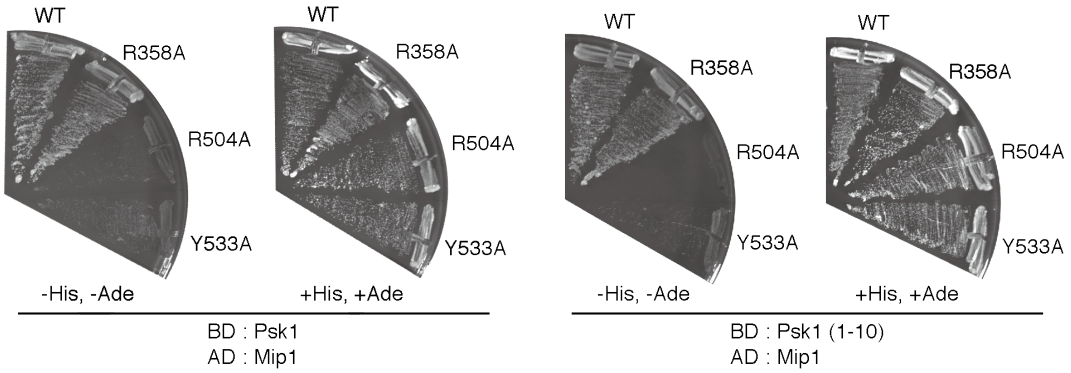

Fig. S3. Mip1 R504A and Y533A mutants showed defective interaction with Psk1 (related to Fig. 4A).

The Mip1 mutants carrying the indicated alanine substitutions were expressed as prey (AD) along with either Psk1 or Psk1 (1-10) as bait (BD) in the budding yeast Y2HGold, and their interaction was tested by adenine and histidine auxotrophy.

S. pombe	Psk1	2	PVFMFDEHD	10
	Atg13	536	EIFDIDTYN	544
S. cerevisiae	Ypk3	1	MIFSLDEEL	9
A. thaliana	ATG13	187	SSFSDIFSG	195
H. sapiens	S6K1	3	GVFDIDLDQ	11
	4EBP1	112	SQFEMDI	118
	PRAS40	127	GLFVMDEDA	135

Fig. S4. Alignment of the TOS motif-like sequences found in fission yeast and budding yeast.

The TOS motif-like sequences of fission yeast Atg13 and budding yeast Ypk3 are aligned with the TOS motif found in fission yeast Psk1 as well as those reported in plant ATG13, and human S6K1, 4EBP1 and PRAS40 (Choi et al., 2003; Nojima et al., 2003; Oshiro et al., 2007; Schalm and Blenis, 2002; Schalm et al., 2003; Son et al., 2018; Wang et al., 2007). The conserved phenylalanine and aspartic acid residues are shown in red.

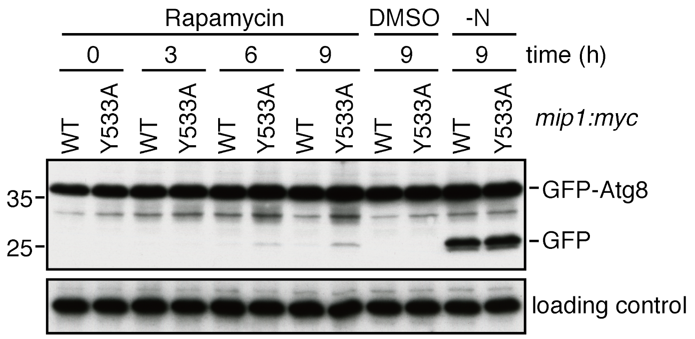

Fig. S5. Autophagy is weakly induced in the *mip1-Y533A* mutant after rapamycin treatment.

The *mip1:myc* and *mip1-Y533A:myc* strains were treated with either DMSO (negative control) or 200 ng/ml of rapamycin in EMM at 30°C for the indicated time. These strains were also incubated in EMM with no nitrogen source at 30°C as positive controls (-N). Their cell lysate was subjected to immunoblotting analysis, and autophagy was monitored by detecting the GFP moiety cleaved from the GFP-Atg8 fusion protein.

Table S1. ***S. pombe*** **strains list used in this study.**

Strain ID	Genotype	Sorce, Reference
CA15458	*h-*	Lab stock
CA7589	*h- leu1-32 Δpsk1::KanMX4*	This study
CA15764	*h- psk1:5FLAG(KanMX6)*	This study
CA15910	*h- psk1V3A:5FLAG(KanMX6)*	This study
CA15911	*h- psk1F4A:5FLAG(KanMX6)*	This study
CA15595	*h- psk1F6A:5FLAG(KanMX6)*	This study
CA15596	*h- psk1D7A:5FLAG(KanMX6)*	This study
CA15594	*h- psk1ΔN10:5FLAG(KanMX6)*	This study
CA7587	*h- Δpsk1::KanMX4*	Bioneer
CA15582	*h- mip1:13myc(KanMX6)*	This study
CA15585	*h- mip1R113A:13myc(KanMX6)*	This study
CA15400	*h- mip1R358A:13myc(KanMX6)*	This study
CA15463	*h- mip1R504A:13myc(KanMX6)*	This study
CA15402	*h- mip1Y533A:13myc(KanMX6)*	This study
CA17794	h^{90} *mip1:13myc(KanMX6)*	This study
CA17798	h^{90} *mip1R113A:13myc(KanMX6)*	This study
CA17795	h^{90} *mip1R358A:13myc(KanMX6)*	This study
CA17796	h^{90} *mip1R504A:13myc(KanMX6)*	This study
CA17806	h^{90} *mip1Y533A:13myc(KanMX6)*	This study
CA15693	*h- mip1:mEGFP:GFP(hph)*	(Chia et al., 2017)
CA15589	*h- mip1Y533A:mEGFP:GFP(hph)*	This study
CA6502	*h- FLAG:tor2(kanR)*	(Hayashi et al., 2007)
CA15087	*h- FLAG:tor2(kanR) mip1:13myc(hph)*	This study
CA15052	*h- FLAG:tor2(kanR) mip1R358A :13myc(hph)*	This study
CA15695	*h- FLAG:tor2(kanR) mip1R504A:13myc(hph)*	This study
CA15054	*h- FLAG:tor2(kanR) mip1Y533A:13myc(hph)*	This study
CA15504	*h- mip1:13myc(hph) psk1:5FLAG(KanMX6)*	This study
CA15720	*h- mip1Y533A:13myc(hph) psk1:5FLAG(KanMX6)*	This study
CA15506	*h- mip1:13myc(hph) Δpsk1::KanMX4*	This study
CA15591	*h- mip1:13myc(hph) psk1ΔN10:5FLAG(KanMX6)*	This study
CA15539	*h- mip1:13myc(KanMX6) sck1:5FLAG(hph)*	This study
CA15469	*h- mip1Y533A:13myc(KanMX6) sck1:5FLAG(hph)*	This study
CA15538	*h- mip1:13myc(KanMX6) sck2:5FLAG(hph)*	This study
CA15470	*h- mip1Y533A:13myc(KanMX6) sck2:5FLAG(hph)*	This study
CA15546	*h- mip1:13myc(KanMX6) maf1:5FLAG(hph)*	This study
CA15550	*h- mip1Y533A:13myc(KanMX6) maf1:5FLAG(hph)*	This study
CA15499	*h- mip1:13myc(hph) atg13:13myc(KanMX6)*	This study

Journal of Cell Science • Supplementary information

CA15503	*h- mip1Y533A:13myc(hph) atg13:13myc(KanMX6)*	This study
CA15543	*h- mip1:13myc(hph)*	This study
CA17566	*h- mip1:13myc(hph) atg13F538A:13myc(KanMX6)*	This study
CA17568	*h- mip1Y533A:13myc(hph) atg13F358A:13myc(KanMX6)*	This study
CA16043	*h- mip1:13myc(hph) P3nmt1(kan):GFP:atg8*	This study
CA16049	*h- mip1Y533A:13myc(hph) P3nmt1(kan):GFP:atg8*	This study
CA17789	*h- atg13:13myc(KanMX6) P3nmt1(kan):GFP:atg8*	This study
CA17785	*h- atg13F538A:13myc(KanMX6) P3nmt1(kan):GFP:atg8*	This study

Journal of Cell Science • Supplementary information
