# Supplementary material for: Fission yeast TOR complex 1 phosphorylates Psk1 through an evolutionarily conserved interaction mediated by the TOS motif
Source: J Cell Sci. 2021 Oct 12;134(19):jcs258865. doi: 10.1242/jcs.258865 (PMC8542387; doi:10.1242/jcs.258865)
Supplement: Reviewer comments [file joces-134-258865_review_history.pdf]

Fission yeast TOR complex 1 phosphorylates Psk1 through an evolutionarily conserved interaction mediated by the TOS motif

Yuichi Morozumi, Ai Hishinuma, Suguru Furusawa, Fajar Sofyantoro, Hisashi Tatebe and Kazuhiro Shiozaki
DOI: 10.1242/jcs.258865

Editor: David Glover

Review timeline

Original submission:	5 May 2021
Editorial decision:	8 June 2021
First revision received:	2 August 2021
Editorial decision:	19 August 2021
Second revision received:	22 August 2021
Accepted:	3 September 2021

Original submission

First decision letter

MS ID#: JOCES/2021/258865

MS TITLE: Fission yeast TOR complex 1 recruits and phosphorylates Psk1 through evolutionarily conserved interaction mediated by the TOS motif

AUTHORS: Yuichi Morozumi, Ai Hishinuma, Suguru Furusawa, Fajar Sofyantoro, Hisashi Tatebe, and Kazuhiro Shiozaki
ARTICLE TYPE: Research Article

We have now reached a decision on the above manuscript.

To see the reviewers' reports and a copy of this decision letter, please go to: https://submit-jcs.biologists.org and click on the 'Manuscripts with Decisions' queue in the Author Area. (Corresponding author only has access to reviews.)

As you will see, the reviewers raise a number of substantial criticisms that prevent me from accepting the paper at this stage. They suggest, however, that a revised version might prove acceptable, if you can address their concerns. If you think that you can deal satisfactorily with the criticisms on revision, I would be pleased to see a revised manuscript. We would then return it to the reviewers.

We are aware that you may be experiencing disruption to the normal running of your lab that makes experimental revisions challenging. If it would be helpful, we encourage you to contact us to discuss your revision in greater detail. Please send us a point-by-point response indicating where you are able to address concerns raised (either experimentally or by changes to the text) and where you will not be able to do so within the normal timeframe of a revision. We will then provide further guidance. Please also note that we are happy to extend revision timeframes as necessary.

Please ensure that you clearly highlight all changes made in the revised manuscript. Please avoid using 'Tracked changes' in Word files as these are lost in PDF conversion.

© 2021. Published by The Company of Biologists under the terms of the Creative Commons Attribution License (https://creativecommons.org/licenses/by/4.0/).

I should be grateful if you would also provide a point-by-point response detailing how you have dealt with the points raised by the reviewers in the 'Response to Reviewers' box. Please attend to all of the reviewers' comments. If you do not agree with any of their criticisms or suggestions please explain clearly why this is so.

Reviewer 1

Advance summary and potential significance to field

Background TOR complex 1 (TORC1) is a multi-subunit protein kinase complex that plays a pivotal role in cell growth and proliferation in response to environmental cues. RAPTOR, a non-catalytic core component of TORC1, is required for substrate recognition; this component recruits various substrates to TORC1, thereby enabling the catalytic subunit to phosphorylate them. In mammalian cells, a subset of substrates including S6 kinase S6K1 and the translation regulator 4EBP1 contains a short peptide sequence, called a TOR signaling (TOS) motif, with which RAPTOR directly interacts. Although TORC1 including RAPTOR is conserved from yeast to human beings, a TOS motif has not been identified in yeast.

Findings In this study, Morozumi et al. have identified a TOS motif in the fission yeast S6 kinase Psk1. The conserved Phe and Asp residues within the TOS motif are essential for interaction between RAPTOR-Mip1 and Psk1 and therefore phosphorylation of Psk1 by TORC1. A point mutation of Mip1 (Mip1-Y533A), in which tyrosine 533 situated in the binding interface between RAPTOR and the TOS motif is mutated to alanine, abolishes interaction between Mip1 and Psk1. Furthermore, the authors have succeeded in identifying another TOS motif in Atg13, which is a known substrate of TORC1.

Advance and Significance This is the first report on the existence of the TOS motif in yeast. This work further underpins the evolutionary conservation of TORC1: the mechanism of substrate recognition.

Comments for the author

This is a nice piece of work. Experiments are properly performed, the data presented are mostly very clear and the text is well written.

Major comments
1) Phenotypic consequences Mip1 mutants, in which tyrosine or arginine critical for interaction between Mip1 and the TOS motif is substituted with alanine, appear defective in TOR signalling, as these mutants display hyper-sensitivity to rapamycin. Have the authors examined other phenotypes such as growth properties or mating efficiency?
As TORC1 inhibits autophagy, it is conceivable that phosphorylation of Atg13 by TORC1 downregulates its function. Does the Atg13 mutant (Atg13-F538A) defective in binding to Mip1 display any phenotypes such as enhanced or promiscuous autophagy?

2) Localisation of Mip1 mutants In Figure 4C, localisation of Mip1-Y533A is shown. How about other mutants such as Mip1-R358A and Mip1-R504A? Do these mutants delocalise from the vacuole?

3) Quantification Many of the data shown are clear-cut, almost all or none, by which quantification would not be necessary. However, those shown in Figure 4B and 4E need more careful assessment. First, how many times were experiments performed? Second, quantification needs to be shown.

Minor comments
4) Materials and Methods The authors need to give more detailed Information. Many strains were constructed, but experimental procedures are not provided. Pho-tag should be mentioned in this section in addition to the figure legend (Figure 5). For cell cultures using nmt1 promoter-driven psk1, how long were cells grown in the absence of thiamine? No detailed explanations of spot tests are given.

© 2021. Published by The Company of Biologists under the terms of the Creative Commons Attribution License (https://creativecommons.org/licenses/by/4.0/).

5) 4EBP1 I guess that 4EBP1 does not exist in yeast. If so, please state this fact in the text.

6) TOS motifs For clarity, amino acid sequence alignment of the TOS motifs found in Atg13 and budding yeast Ypk3 would be better to be shown, maybe as Supplementary Information.

7) protein size markers The position of size markers needs to be shown in the data using SDS-PAGE gels.

8) line 585
'were' should be 'was'.

Reviewer 2

Advance summary and potential significance to field

In the paper titled "Fission yeast TOR complex 1 recruits and phosphorylates Psk1 through evolutionary conserved interaction mediated by the TOS motif", Morozumi Y. et al. describe a TOR signaling (TOS) motif in Psk1, the fission yeast ortholog of S6K. The TOS motif at the N-terminus of Psk1 is required for Mip1 binding and for TORC1 phosphorylation at Psk1-T415. Moreover, the authors identify a TOS motif in Atg13 by screening phosphorylation level of TORC1 substrates under TOS deficient mip1-Y533A background. The study for the first time demonstrates the existence of TOR signaling (TOS) motif dependent TORC1 substrate recruitment in lower eukaryotes. Moreover, the 5 residues TOS motif sequence of Psk1 slightly diverges from the canonical TOS motifs of human S6K1, 4E-BP1 and PRAS40, even though TOS motif binding residues in RAPTOR are conserved in Mip1. This study hints to a wider definition of the consensus TOS motif sequence, and might allow identification of TOS motifs in other mTORC1 substrates, for which no apparent TOS motifs have been identified.

Comments for the author

The paper is well written and clearly structured. The data is of high quality and the results are very convincing. However, the study fails to demonstrate a functional phenotype of TOS motif mutations Psk1-P4A or Atg13-P538A. Additional experiments are needed to demonstrate the necessity of the TOS motif of Atg13 for the activation of autophagy. In Figure 5, the authors conclude that alanine substitution of residue Y533 in Mip1 (as well as residue F538 in Atg13) impair TORC1-dependent phosphorylation of Atg13. However, they fail to show a functional consequence of this. The authors should assess whether autophagy induction is impaired in cell expressing Mip1-Y533A and in cells expressing Atg13-P538A.

[Editorial note: reviewer is referring to F4A and F538A mutations in this section.]

Minor point
Line 105: According to the reference Nakashima et al. TORC1 phosphorylates Psk1 at T392, not at T389.
Potential extension of the study
Regarding Figure 5. Based mainly on loss of function experiments (i.e., mutation to alanine of key residues in the TOS motif) the authors demonstrate that Psk1 contains a TOS motif. Although convincing, the authors' conclusion would be reinforced if they substituted a region of Maf1, Sck1, or Sck2 (ideally the Mip1 binding region, if known) with a region of Psk1 containing the TOS-motif and assessed the phosphorylation behavior of the resulting chimeric proteins. One would expect the phosphorylation of the resulting chimeric proteins to depend on Y533A in Mip1. A similar approach has been recently described by the Ballabio lab with TFEB and S6K (Napolitano G, et al. Nature. 585, 597-602 (2020). Figures 2C-E).

First revision

Author response to reviewers' comments

© 2021. Published by The Company of Biologists under the terms of the Creative Commons Attribution License (https://creativecommons.org/licenses/by/4.0/).

Point-by-point response

Reviewer 1

Comments for the Author:

This is a nice piece of work. Experiments are properly performed, the data presented are mostly very clear and the text is well written.

Major comments
1) Phenotypic consequences
Mip1 mutants, in which tyrosine or arginine critical for interaction between Mip1 and the TOS motif is substituted with alanine, appear defective in TOR signalling, as these mutants display hyper-sensitivity to rapamycin. Have the authors examined other phenotypes such as growth properties or mating efficiency?

As shown in new Figure S2, we have assessed the growth rate and sexual differentiation of the *mip1* mutant strains constructed in this study, and found no significant difference from those of the wild-type strain.

As TORC1 inhibits autophagy, it is conceivable that phosphorylation of Atg13 by TORC1 downregulates its function. Does the Atg13 mutant (Atg13-F538A) defective in binding to Mip1 display any phenotypes such as enhanced or promiscuous autophagy?

The autophagy phenotypes of the *mip1-Y533A* and *atg13-F538A* mutants have been examined by monitoring the autophagic cleavage of the GFP moiety from the GFP-Atg8 fusion protein (p.13 of the revised manuscript). Autophagy was induced only after nitrogen starvation in wild-type, *mip1-Y533A* and *atg13-F538A* cells, but not before starvation, suggesting that the *mip1-Y533A* and *atg13-F538A* mutations are not enough to induce autophagy. Rapamycin induced a low level of released GFP in the *mip1-Y533A* mutant (new Fig. S5), but not in the wild-type and *atg13-F538A* mutant strains. These results indicate that the lack of the TOS motif-dependent phosphorylation of Atg13 *per se* is insufficient for the autophagy initiation, and there may be additional TORC1 substrate(s) involved in the negative regulation of autophagy.

2) Localisation of Mip1 mutants
In Figure 4C, localisation of Mip1-Y533A is shown. How about other mutants such as Mip1-R358A and Mip1-R504A? Do these mutants delocalise from the vacuole?

We have examined the cellular localization of Mip1-R358A and Mip1-R504A in addition to Mip1-Y533A, as shown in new Figure 4C, and found that all three mutant forms of Mip1 showed vacuolar localization similar to that of wild-type Mip1.

3) Quantification
Many of the data shown are clear-cut, almost all or none, by which quantification would not be necessary. However, those shown in Figure 4B and 4E need more careful assessment. First, how many times were experiments performed? Second, quantification needs to be shown.

We have performed the quantification analyses as suggested. In new Figure 4B, the Mip1 levels in the immunopurified fractions were quantified and their mean±s.d. values from three independent experiments are shown in a bar graph. Similarly, the mean±s.d. (n=3, independent experiments) of the quantified phospho-4EBP1 level is shown as a value relative to that of the WT control in new Figure 4E.

Minor comments
4) Materials and Methods
The authors need to give more detailed Information. Many strains were constructed, but experimental procedures are not provided. Pho-tag should be mentioned in this section in addition to the figure legend (Figure 5). For cell cultures using nmt1 promoter-driven psk1, how long were cells grown in the absence of thiamine? No detailed explanations of spot tests are given.

We have included more detailed information in the Materials and Methods section as advised by the reviewer (p.18-20).

© 2021. Published by The Company of Biologists under the terms of the Creative Commons Attribution License (https://creativecommons.org/licenses/by/4.0/).

5) 4EBP1
I guess that 4EBP1 does not exist in yeast. If so, please state this fact in the text.

We have included such a description accordingly (line 225).

6) TOS motifs
For clarity, amino acid sequence alignment of the TOS motifs found in Atg13 and budding yeast Ypk3 would be better to be shown, maybe as Supplementary Information.

As suggested by the reviewer, we have added an amino acid sequence alignment of the TOS motifs found in Atg13 and budding yeast Ypk3 with the known TOS motifs in new Figure S4.

7) protein size markers
The position of size markers needs to be shown in the data using SDS-PAGE gels.

The position of size markers has been included in each data using SDS-PAGE.

8) line 585
'were' should be 'was'.

We have corrected the typo (line 647 in the revised manuscript).

Reviewer 2
Comments for the Author:
The paper is well written and clearly structured. The data is of high quality and the results are very convincing. However, the study fails to demonstrate a functional phenotype of TOS motif mutations Psk1-P4A or Atg13-P538A. Additional experiments are needed to demonstrate the necessity of the TOS motif of Atg13 for the activation of autophagy. In Figure 5, the authors conclude that alanine substitution of residue Y533 in Mip1 (as well as residue F538 in Atg13) impair TORC1-dependent phosphorylation of Atg13. However, they fail to show a functional consequence of this. The authors should assess whether autophagy induction is impaired in cell expressing Mip1-Y533A and in cells expressing Atg13-P538A.

[Editorial note: reviewer is referring to F4A and F538A mutations in this section.]

We have assessed the Psk1-dependent phosphorylation of Rps6 (Nakashima et al., 2012, cited in the manuscript), in the *psk1* mutant strains, including *psk1-F4A*. As shown in new Figure 2C, the phosphorylation of Rps6 was significantly compromised in all the *psk1* mutants that are defective in the TORC1-dependent phosphorylation of Psk1. This result indicates that the TOS motif-dependent phosphorylation of Psk1 by TORC1 is a critical step for the activation of Psk1.

The autophagy phenotypes of the *mip1-Y533A* and *atg13-F538A* mutants have been examined by monitoring the autophagic cleavage of the GFP moiety from the GFP-Atg8 fusion protein (p.13 of the revised manuscript). Autophagy was induced only after nitrogen starvation in wild-type, *mip1-Y533A* and *atg13-F538A* cells, but not before starvation, suggesting that the *mip1-Y533A* and *atg13-F538A* mutations are not enough to induce autophagy. Rapamycin induced a low level of released GFP in the *mip1-Y533A* mutant (new Fig. S5), but not in the wild-type and *atg13-F538A* mutant strains. These results indicate that the lack of the TOS motif-dependent phosphorylation of Atg13 *per se* is insufficient for the autophagy initiation, and there may be additional TORC1 substrate(s) involved in the negative regulation of autophagy.

Minor point
Line 105: According to the reference Nakashima et al. TORC1 phosphorylates Psk1 at T392, not at T389.

We have corrected the typo (line 105).

Potential extension of the study
Regarding Figure 5. Based mainly on loss of function experiments (i.e., mutation to alanine of key residues in the TOS motif) the authors demonstrate that Psk1 contains a TOS motif. Although convincing, the authors' conclusion would be reinforced if they substituted a region of Maf1, Sck1,

© 2021. Published by The Company of Biologists under the terms of the Creative Commons Attribution License (https://creativecommons.org/licenses/by/4.0/).

or Sck2 (ideally the Mip1 binding region, if known) with a region of Psk1 containing the TOS-motif and assessed the phosphorylation behavior of the resulting chimeric proteins. One would expect the phosphorylation of the resulting chimeric proteins to depend on Y533A in Mip1. A similar approach has been recently described by the Ballabio lab with TFEB and S6K (Napolitano G, et al. Nature. 585, 597-602 (2020). Figures 2C-E).

We have fused the N-terminal 10 amino acid residues of Psk1, which include the TOS motif, to the Sck1 N-terminus and examined the phosphorylation of the chimeric protein; the fusion of the Psk1 TOS motif to Sck1 shows little impact on its phosphorylation both in wild-type and *mip1-Y533A* mutant strains (data not shown). This is not surprising, as Sck1 is likely to have its own mechanism to be recognized by Mip1. Thus, to perform the experiments suggested by the reviewer, it is necessary to replace the Mip1-binding region in Sck1 with the Psk1 TOS motif. Unfortunately, the Mip1-binding region is currently unknown for Sck1, Sck2 and Maf1.

Second decision letter

MS ID#: JOCES/2021/258865

MS TITLE: Fission yeast TOR complex 1 phosphorylates Psk1 through evolutionarily conserved interaction mediated by the TOS motif

AUTHORS: Yuichi Morozumi, Ai Hishinuma, Suguru Furusawa, Fajar Sofyantoro, Hisashi Tatebe, and Kazuhiro Shiozaki
ARTICLE TYPE: Research Article

We have now reached a decision on the above manuscript.

To see the reviewers' reports and a copy of this decision letter, please go to: https://submit-jcs.biologists.org and click on the 'Manuscripts with Decisions' queue in the Author Area. (Corresponding author only has access to reviews.)

As you will see, the reviewers gave favourable reports but raised some critical points that will require amendments to your manuscript. I hope that you will be able to carry these out because I would like to be able to accept your paper, depending on further comments from reviewers.

We are aware that you may be experiencing disruption to the normal running of your lab that makes experimental revisions challenging. If it would be helpful, we encourage you to contact us to discuss your revision in greater detail. Please send us a point-by-point response indicating where you are able to address concerns raised (either experimentally or by changes to the text) and where you will not be able to do so within the normal timeframe of a revision. We will then provide further guidance. Please also note that we are happy to extend revision timeframes as necessary.

Please ensure that you clearly highlight all changes made in the revised manuscript. Please avoid using 'Tracked changes' in Word files as these are lost in PDF conversion.

I should be grateful if you would also provide a point-by-point response detailing how you have dealt with the points raised by the reviewers in the 'Response to Reviewers' box. Please attend to all of the reviewers' comments. If you do not agree with any of their criticisms or suggestions please explain clearly why this is so.

Reviewer 1

Advance summary and potential significance to field

© 2021. Published by The Company of Biologists under the terms of the Creative Commons Attribution License (https://creativecommons.org/licenses/by/4.0/).

Background TOR complex 1 (TORC1) is a multi-subunit protein kinase complex that plays a pivotal role in cell growth and proliferation in response to environmental cues. RAPTOR, a non-catalytic core component of TORC1, is required for substrate recognition; this component recruits various substrates to TORC1, thereby enabling the catalytic subunit to phosphorylate them. In mammalian cells, a subset of substrates including S6 kinase S6K1 and the translation regulator 4EBP1 contains a short peptide sequence, called a TOR signaling (TOS) motif, with which RAPTOR directly interacts. Although TORC1 including RAPTOR is conserved from yeast to human beings, a TOS motif has not been identified in yeast.

Findings In this study, Morozumi et al. have identified a TOS motif in the fission yeast S6 kinase Psk1. The conserved Phe and Asp residues within the TOS motif are essential for interaction between RAPTOR-Mip1 and Psk1 and therefore phosphorylation of Psk1 by TORC1. A point mutation of Mip1 (Mip1-Y533A), in which tyrosine 533 situated in the binding interface between RAPTOR and the TOS motif is mutated to alanine, abolishes interaction between Mip1 and Psk1. Furthermore, the authors have succeeded in identifying another TOS motif in Atg13, which is a known substrate of TORC1.

Advance and Significance This is the first report on the existence of the TOS motif in yeast. This work further underpins the evolutionary conservation of TORC1: the mechanism of substrate recognition.

Comments for the author

In this revised manuscript, the authors addressed several points that I raised in my previous review. For quantification (Figure 4B and E), p-values should be shown. As mean ± s.d. values are obtained, this would be straightforward.
For mating assays (Figure S2), the authors did not use conditions that prevent mating: i. e. mating assay using the media containing nitrogen. Instead, they used standard nitrogen-depleted media in which even 80% of wild-type cells conjugate. The mip1 mutants are hypomorphic and therefore, I expected that they would exhibit a precocious mating behaviour like some tor2 mutants; mating occurs in the presence of nitrogen. Mating assays, therefore, should have been performed on plates containing normal growth media. This is the point which I am disappointed with in this revised manuscript. As I did not describe a specific condition for mating assays in my previous evaluation, I shall not ask again for additional experiments. But it is a pity that the authors could not find any physiological defects in the mip1 mutants that display compromised interaction with the TOS motif or the Tor2 catalytic subunit.

<u>Reviewer 2</u>

Advance summary and potential significance to field

In the revised manuscript “Fission yeast TOR complex 1 phosphorylates Psk1 through evolutionarily conserved interaction mediated by the TOS motif”, Morozumi Y. et al. expand their study with several experiments. The authors show that TOS-motif targeted interference with the phosphorylation of Psk1 reduces the phosphorylation of its target Rsp6, and that growth properties and sexual differentiation was unaltered in mip1 mutant strains. Moreover, the authors assessed induction of autophagy of the mip1-Y533A and atg13-F538A strains using a GFP-Atg8 autophagic degradation assay.

All points raised during review of the initial submission were addressed, and I recommend the manuscript for publication.

Comments for the author

None

© 2021. Published by The Company of Biologists under the terms of the Creative Commons Attribution License (https://creativecommons.org/licenses/by/4.0/).

Second revision

Author response to reviewers' comments

Point-by-point response

Reviewer 1 Comments for the Author:
In this revised manuscript, the authors addressed several points that I raised in my previous review.
For quantification (Figure 4B and E), p-values should be shown. As mean s.d. values are obtained, this would be straightforward.

As advised by the reviewer, we have calculated p-values using Student's t-test and revised Figure 4B and E. *P*<0.05 is considered statistically significant.

For mating assays (Figure S2), the authors did not use conditions that prevent mating: i.e. mating assay using the media containing nitrogen. Instead, they used standard nitrogen-depleted media in which even 80% of wild-type cells conjugate. The mip1 mutants are hypomorphic and therefore, I expected that they would exhibit a precocious mating behaviour like some tor2 mutants; mating occurs in the presence of nitrogen. Mating assays, therefore, should have been performed on plates containing normal growth media. This is the point which I am disappointed with in this revised manuscript. As I did not describe a specific condition for mating assays in my previous evaluation, I shall not ask again for additional experiments. But it is a pity that the authors could not find any physiological defects in the mip1 mutants that display compromised interaction with the TOS motif or the Tor2 catalytic subunit.

We have examined sexual differentiation of the *mip1* mutant strains on rich yeast extract medium, and found that no precocious mating occurred in those mutants, in contrast to some of the *tor2* mutants reported previously. We have included this new result in the revised manuscript (lines 190-193).

Third decision letter

MS ID#: JOCES/2021/258865

MS TITLE: Fission yeast TOR complex 1 phosphorylates Psk1 through evolutionarily conserved interaction mediated by the TOS motif

AUTHORS: Yuichi Morozumi, Ai Hishinuma, Suguru Furusawa, Fajar Sofyantoro, Hisashi Tatebe, and Kazuhiro Shiozaki
ARTICLE TYPE: Research Article

I am happy to tell you that your manuscript has been accepted for publication in Journal of Cell Science, pending standard ethics checks.

© 2021. Published by The Company of Biologists under the terms of the Creative Commons Attribution License (https://creativecommons.org/licenses/by/4.0/).
